# Elucidating the Role of Cerebellar Synaptic Dysfunction in *C9orf72*-ALS/FTD — a Systematic Review and Meta-Analysis

**DOI:** 10.1007/s12311-021-01320-0

**Published:** 2021-09-07

**Authors:** Aleksandra Kaliszewska, Joseph Allison, Tarik-Tarkan Col, Christopher Shaw, Natalia Arias

**Affiliations:** 1 UK Dementia Research Institute At King’s College London, Institute of Psychiatry, Psychology and Neuroscience, Department of Basic & Clinical Neuroscience, Maurice Wohl Clinical Neuroscience Institute, 5 Cutcombe road, Camberwell, SE59RX London UK; 2 Centre for Brain Research, University of Auckland, 85 Grafton Road, Auckland, 1023 New Zealand; 3 INEUROPA, Instituto de Neurociencias del Principado de Asturias, Plaza Feijoo s/n, 33003 Oviedo, Spain

**Keywords:** *C9orf72*, Synaptic dysfunction, Cerebellum, ALS, FTD

## Abstract

A hexanucleotide repeat expansion in the *C9orf72* gene is the most common genetic cause of amyotrophic lateral sclerosis (ALS) and frontotemporal dementia (FTD) with synaptic dysfunction identified as an early pathological hallmark. Although TDP-43 pathology and overt neurodegeneration are largely absent from the cerebellum, the pathological hallmarks of RNA foci and dipeptide repeat protein (DPR) inclusions are most abundant. Here, we present a systematic literature search in the databases of PubMed, Scopus, Embase, Web of Science and Science Direct up until March 5, 2021, which yielded 19,515 publications. Following the exclusion criteria, 72 articles were included having referred to *C9orf72*, synapses and the cerebellum. Meta-analyses were conducted on studies which reported experimental and control groups with means and standard deviations extracted from figures using the online tool PlotDigitizer. This revealed dendritic defects (*P* = 0.03), reduced *C9orf72* in human patients (*P* = 0.005) and DPR-related neuronal loss (*P* = 0.0006) but no neuromuscular junction abnormalities (*P* = 0.29) or cerebellar neuronal loss (*P* = 0.23). Our results suggest that dendritic arborisation defects, synaptic gene dysregulation and altered synaptic neurotransmission may drive cerebellar synaptic dysfunction in C9-ALS/FTD. In this review, we discuss how the chronological appearance of the different pathological hallmarks alters synaptic integrity which may have profound implications for disease progression. We conclude that a reduction in *C9orf72* protein levels combined with the accumulation of RNA foci and DPRs act synergistically to drive C9 synaptopathy in the cerebellum of C9-ALS/FTD patients.

## Introduction

The most common genetic cause of both amyotrophic lateral sclerosis (ALS) and frontotemporal dementia (FTD) has been proven to be a large hexanucleotide repeat expansion (G_4_C_2_)_n_ within intron 1 of *C9orf72* (C9) [1, 2]. As a result of the expansion, three pathogenic mechanisms have been proposed as the underlying cause of C9-ALS/FTD: (1) loss of function due to G4C2 repeat expansion leading to downregulation of *C9orf72* protein expression; (2) toxic gain of function by recruitment of other RNA-binding proteins into G_4_C_2_ RNA foci; and (3) the non-ATG initiated RAN translation of RNA repeats, which results in the production of toxic dipeptide protein repeat (DPRs) [3–5].

The cerebellum is home to approximately 80% of all neurons in the human brain, which mediate reciprocal connections with multiple regions throughout the brain and spinal cord [6, 7]. Importantly, Renton et al. (2011) [2] detected the highest expression level of *C9orf72* RNA within the cerebellum of neuropathologically normal individuals. This finding is relevant as the cerebellum executes a major role in regulating sensorimotor control and higher order cognitive functions such as gait, coordination and fine balance, as well as spatial memory, apathy and executive control — all of which can be impaired in patients diagnosed with C9-ALS/FTD [8, 9]. However, this dysfunction is ascribed to frontal lobe pathology, and the cerebellum has been largely overlooked as a region of interest in patients with ALS/FTD, despite key findings suggesting the relevance of this brain region. In this review, we want to focus on the roles played by decreased *C9orf72* protein, RNA foci and DPRs in displaying different toxic properties in distinct animal and cellular models [10–15] and specifically in developing cerebellar synaptic dysfunction.

*C9orf72* protein is predominantly localised to the pre-synaptic and post-synaptic compartments in the mouse brain [16, 17]. Xiao et al. (2019) have shown that *C9orf72* is present in synapses of the granular layer of the cerebellum when comparing C9-wild type versus C9-knockout animals [17], resulting in the suggestion that it may be involved in synaptic transmission and autophagy [16–19]. Downregulation of *C9orf72* impairs autophagy and may contribute to the accumulation of the transactive response DNA-binding protein 43 kDa (TDP-43) and p62 [18]. However, a distinct characteristic of C9-ALS/FTD is identifiable in the spatial segregation of TDP-43 and p62 proteinaceous inclusions, which are most abundant in the cerebellum [20].

The role of C9 RAN-translated DPRs in synaptic dysfunction was illustrated by Xu and Xu (2018) who induced the expression of different DPRs in *Drosophila* models [21]. They observed that poly-GR and poly-PR overexpressing flies presented altered synaptic boutons at neuromuscular junctions (NMJs). In contrast, Jensen et al. (2020) [4] observed that poly-GA aggregates are located in neurites and are less mobile at longer length repeats (400 compared to 50 repeats). Moreover, the authors found that poly-GA causes reductions in synaptic vesicle-associated protein 2 (SV2), alters Ca^2+^ influx and inhibits synaptic vesicle release resulting in earlier iPSC death [4]. In addition, the presence of DPRs has been linked to marked reductions in dendritic spine densities and overall dendritic arborisation in both in vitro and in vivo models [22–24].

Indeed, May et al. (2014) [22] have shown that overexpression of poly-GA in primary neuronal cultures caused severe reductions in dendritic arborisation due to the co-aggregation and sequestration of Unc119, a protein also known to suppress axonal arborisation. In another study by Park et al. (2020) [24], the most significant reduction in dendritic branches was associated with the presence of arginine-rich DPRs (PR and GR) in *C9orf72 Drosophila* neurons. Moreover, Schweizer-Burguete et al. (2015) [23] showed that the overexpression of 48 × GGGGCC repeat RNA (G4C2-48) caused dendritic branching defects and decreased synaptic densities in rodent spinal cord neurons.

Interestingly, there is evidence supporting DPR aggregation in cerebellar tissues of C9-ALS/FTD patients [25–29]. Several studies have documented that poly-GA and poly-GP DPR aggregates predominate in the cerebellum of C9-ALS/FTD patients and may contribute to disease progression [11, 27, 29–32]. Indeed, Zhang et al. (2014) [28] have shown that in primary mouse neuronal cultures, the overexpression of poly-GA leads to the upregulation of cytoplasmic p62-immunopositive inclusions within the granule cell layer of the cerebellum in the absence of neurodegeneration. Moreover, in vivo green fluorescent protein (GFP) tagged mouse models overexpressing poly-GA (GFP-GA_50_), demonstrating more severe neuronal cell loss in the Purkinje layer of the cerebellum which were associated to the aggregation and sequestration of HR23 proteins, responsible for normal proteasome degradation and nucleocytoplasmic transport functions [33]. Conversely, there are conflicting studies where, despite detecting significant poly-GA, GP and GR inclusions in the cerebellar tissues of *C9orf72* patients, no sign of neurodegeneration in the cerebellum, cognitive decline or clinical phenotypes have been found [30–32, 34, 35].

**Fig. 1:**
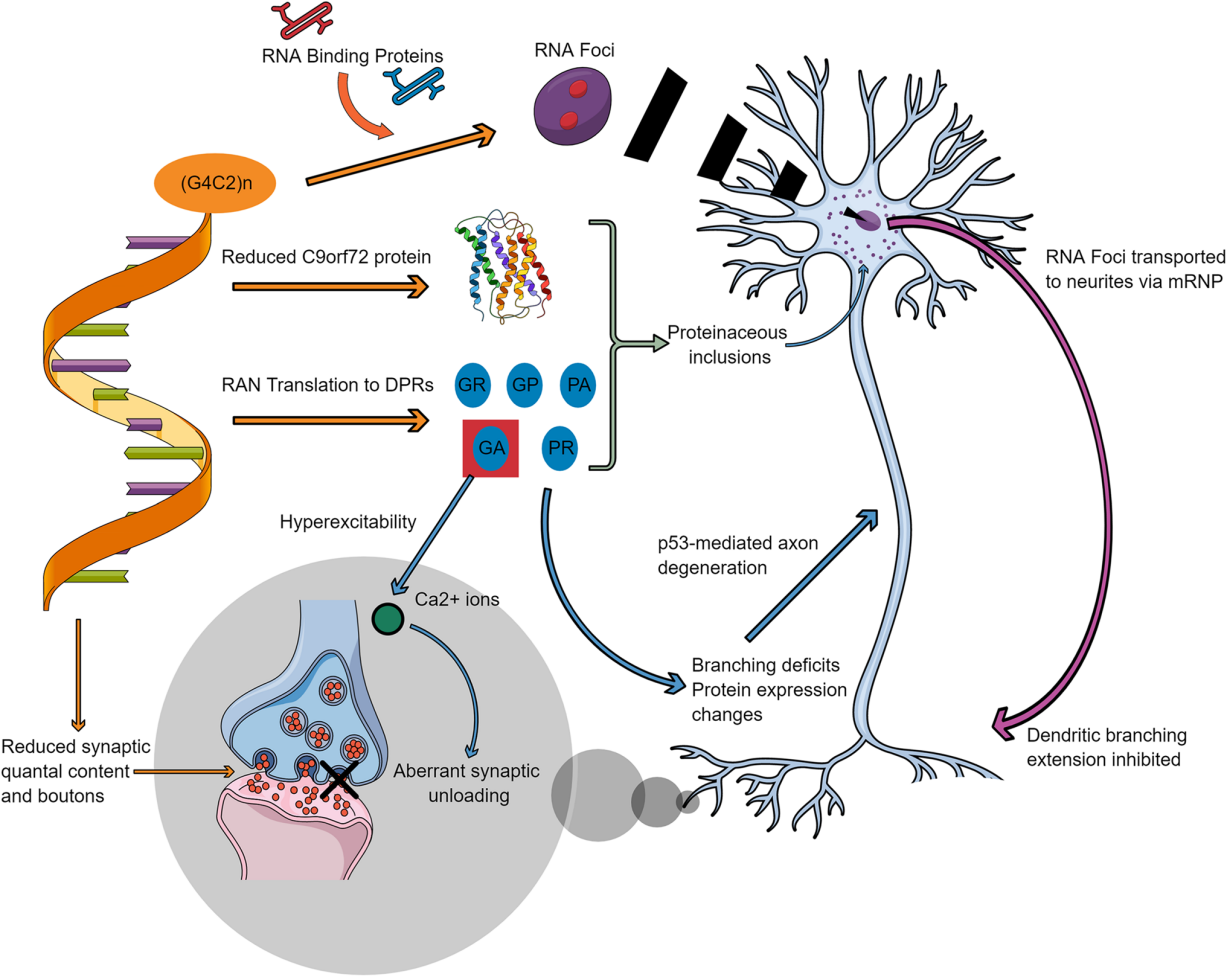
Putative mechanisms underlying synaptic dysfunction in C9orf72-ALS/FTD. A schematic detailing the role of the hexanucleotide expansion, (G_4_C_2_)_n_, of the *C9orf72* gene in driving synaptic, axonal and dendritic dysfunction. This operates through the three main pathogenic mechanisms implicated in C9-ALS/FTD which are haploinsufficiency of the C9orf72 protein and the accumulation of RNA foci and dipeptide repeats (DPRs). Abbreviations: *p53*, tumour protein p53; *RAN*, repeat-associated non-AUG; *Ca*^*2*+^, calcium ions; *mRNP*, messenger ribonucleoprotein; *RNA*, ribonucleic acid

Furthermore, RNA foci are also frequently identified in the molecular and granular cell layers of the cerebellum, where intranuclear foci were significantly larger (~ 500 nm) in comparison to the neocortex (~ 200 nm) in both in vitro and zebrafish models. This has been suggested to be linked to caspase-3-initiated mechanisms of apoptotic neurodegeneration [36]. Interestingly, a more recent clinico-pathological study examining cerebellar and frontal cortical post-mortem tissue from *C9orf72* expansion mutation carriers identified the largest RNA foci burden levels in the Purkinje cells of the cerebellum (~ 70%) compared to all other regional tissue types, without any cerebellar neuronal loss [37]. All these results could suggest a synergistic combination of RNA foci and DPR accumulation which could be underlying cerebellar synaptic dysfunction usually overlooked in C9-ALS/FTD patients (overviewed in Fig. 1).

In the present systematic review, we will examine the most recent literature for cerebellar synaptic dysfunction in *C9orf72* gene mutation carriers of ALS/FTD. We will discuss alterations in neuronal morphology, including structural and functional changes to synapses, deterioration in dendritic morphology and axonal degeneration. Finally, we will address the role of DPRs and RNA foci and whether these pathological features precede cerebellar neuronal dysfunction during the course of gradual neurodegeneration in the cerebellum of C9-ALS/FTD patients.

## Methods

### Literature Search

A systematic literature search was conducted in accordance to the Preferred Reporting Items for Systematic Reviews and Meta-Analyses (PRISMA) guidelines [38]. Original research articles and reviews pertaining to cerebellar synaptic dysfunction in C9-ALS/FTD have been independently searched for in five electronic databases — PubMed, Web of Science (WoS), Scopus, Science Direct and EMBASE. The search was performed by three researchers (NA, JA, AK) on March 5, 2021, using the following search terms and combinations: [“cerebellum” AND “synaptic” AND “C9ORF72”], [“cerebellum” AND “synapsis” AND “C9ORF72”], [“cerebellum” AND “pruning” AND “C9ORF72”], [“cerebellum” AND “dendrites” AND “C9ORF72”], [“cerebellum” AND “neuronal loss” AND “C9ORF72”], [“cerebellum” AND “axonal” AND “C9ORF72”], [“cerebellum” AND “neuron” AND “C9ORF72”], [“cerebellum” AND “C9ORF72” AND “ALS” “cerebellum” AND “C9ORF72” AND “ALS” OR “Amyotrophic lateral sclerosis”], [“cerebellum” AND “C9ORF72” AND “FTD” OR “frontotemporal dementia”], [“synaptic” AND “C9ORF72”], [“dendrites” AND “C9ORF72”], [“axon” AND “C9ORF72”], [“neuronal loss” AND “C9ORF72”]; [“neuronal degeneration” AND “C9ORF72”], [“neuron” AND “C9ORF72” AND “ALS” OR “amyotrophic lateral sclerosis”] and [“neuron” AND “C9ORF72” AND “FTD” OR “Frontotemporal dementia”]. No chronological, language or methodological filters have been imposed on the search engines, and all resulting data sets were exported and compiled in an Excel document. The search strategy was further broadened to include screening of references cited in relevant review articles.

### Study Selection

Following the removal of duplicates, all remaining articles had their titles and abstracts screened for eligibility. Epidemiological studies and articles which did not specifically pertain to *C9orf72* mutation in ALS-FTD were deemed ineligible. After the initial screening phase, full texts of selected studies were retrieved and reviewed in detail against the inclusion criteria. In order for a study to be included in the systematic review, it had to (i) show clear evidence of either synaptic dysfunction or findings relating to *C9orf72* protein/ DPR aggregates/RNA foci, (ii) employ genetic models of *C9orf72* mutation and/ or C9-ALS/FTD patient samples and (iii) examine cerebellum tissue or present findings which can be extrapolated to cerebellar synaptic pathology.

### Meta-Analysis

A continuous random effects model with a standard mean difference was employed to conduct the meta-analysis. Publications that reported (i) dendritic arborisation defects, (ii) NMJ abnormalities, (iii) alterations in neurite length, (iv) reductions in C9orf72 protein, (v) cerebellar neuronal loss and (vi) DPR-related neuronal loss underwent methodological quality assessment performed by two independent researchers to minimise the risk of bias. Studies were excluded from meta-analysis for not reporting the mean, standard deviation (SD) or sample size such as Zhang et al*.* (2014) [28] and lack of quantitative analysis such as Lee et al*.* (2017) [39]. Additionally, G_4_C_2_related neuronal loss was not statistically assessed due to several factors (RNA foci, DPRs, reduced C9orf72) having a potential role in neuronal loss. Significance played no role in the selection process, with studies reporting null findings included by the experimenters. Authors of the relevant publications were not contacted directly regarding the raw data sets. Instead, numerical data was extracted directly from the figures using the online data extractor tool PlotDigitizer. Information regarding the figures used to calculate the different outcomes of meta-analysis is summarised in Table 1. Means, standard deviations and sample sizes were entered into Review Manager [40] which automatically calculated standard mean difference (SMD), confidence intervals (CIs), heterogeneity and overall effect size using a random effects model. Studies were weighted in the final analysis based on the precision of their data as determined by confidence intervals, with greater weights usually indicative of larger sample sizes.

**Table 1:** Overview of the studies included for meta-analysis

Study	Included?	Figure chosen	Relevant meta-analysis	Comment
Herranz-Martin et al. [111]	Yes	Fig. S2D	Cerebellar neuronal loss	Purkinje cell counts between HRE-10 (disease control) and HRE-102
Tan et al. [93]	Yes	Table 3	Cerebellar neuronal loss	Spino- and Cerebro-cerebellum were averaged for Purkinje cells — other studies did not state a specific area of the cerebellum; therefore, both were included to avoid bias
Hao et al. [56]	Yes	Figure 5D	Cerebellar neuronal loss; DPR-related neuronal loss	Purkinje counts across an age range (averaged over time) comparing poly-PR with controls. Figure 5D — molecular layer thickness was not chosen as it is not directly neuronal counts (i.e. neuronal density could be increased in a smaller area)
Zhang et al*.* [69]	Yes	Fig. S3G	Cerebellar neuronal loss; DPR-related neuronal loss	Purkinje cell counts between GFP and poly-PR. Time points were averaged
May et al. [22]	Yes	Figure 3B	Dendritic arborisations	Number of dendritic crossings. All distances from the soma of dendritic crossings were averaged to give a total effect across the neuron
Park et al. [24]	Yes	Figure 1B	Dendritic arborisations	Number of dendritic branch points under different DPR transgenes (DPR effect was averaged)
Schweizer Burguete et al. [23]	Yes	Fig. S4B	Dendritic arborisations	Shows late control versus late experimental of dendritic crossings. Preferred to Fig. 3H due to similarity to May 2014
*Perry *et al*.* [44]	*Yes*	*Figure **2J*	*Dendritic arborisations*	*Percentage retractions of synapses at the NMJ. Means were not multiplied by − 1 as, unlike other studies, a positive increase was reflective of a negative effect*
*O’Rourke *et al*.* [99]	*Yes*	*Figure **3G*	*Dendritic arborisations; NMJ abnormalities*	*Percentage of fragmented NMJs. Means were not multiplied by − 1 as, unlike other studies, a positive increase was reflective of a negative effect*
LaClair et al. [80]	Yes	Figure 2E/F	DPR-related neuronal loss	Figure 2E (GA) and F (PR) were averaged to give an overall DPR effect on hippocampal neuron density as well as to avoid bias regarding which DPR was the most important
Darling et al. [77]	Yes	Figure 1A	DPR-related neuronal loss	All DPRs were averaged to compare against the control. Cell viability of iPSCs was assessed
Zhang et al. [65]	Yes	Figure 1G	DPR-related neuronal loss	Score of hippocampal neuronal loss — time points were averaged
Xu and Xu [21]	Yes	Figure 5E	NMJ abnormalities	Total bouton counts
Perry et al*.* [44]	Yes	Figure 1D	NMJ abnormalities	Total bouton counts. (G_4_C_2_)_8_ was averaged as a control, whilst (G_4_C_2_)_58_ and GR_36/100_ were averaged as experimental
Freibaum et al. [42]	Yes	Figure 1F	NMJ abnormalities	Total bouton counts — control and (G_4_C_2_)_8_ were averaged as this is to represent disease control
*Herranz-Martin *et al*.* [111]	*Yes*	*Figure **3B*	*NMJ abnormalities*	*Percentage of pathological NMJs* — *not included for dendrites as pathological is ambiguous. HRE-10 was averaged with control. Means were not multiplied by − 1 as, unlike other studies, a positive increase was reflective of a negative effect*
Frick et al. [16]	Yes	Figure 6B	Reduced C9orf72	Normalised C9orf72 levels in the cerebellum
Saberi et al*.* [96]	Yes	Figure 5N	Reduced C9orf72	Normalised C9orf72 levels in the frontal cortex (chosen over occipital cortex)
Belzil et al. [103]	Yes	Figure 1B	Reduced C9orf72	Normalised C9orf72 levels in the frontal cortex (chosen over CB due to frontal cortex being more widely studied)
Waite et al. [92]	Yes	Figure 3B	Reduced C9orf72	Normalised C9orf72 levels in the frontal cortex (C9-ALL was chosen over specific variants)
Yang et al. [76]	No	Figure 2B	Dendritic arborisations	Not included as only the number of neurons counted were given, not the number of animals per genotype (such as other studies) which would skew the weighting
Park et al*.* [24]	No	Figure 1C	Neurite length	Dendritic length — *N* not high enough for meta-analysis of neurite length
Zhang et al. [28]	No	Fig. S5B	Neurite length	No sample sizes were given — unable to calculate confidence intervals
Swaminathan et al*.* [73]	No	Figure 5B	Neurite length	Neurite length meta-analysis *N* was too small

Studies that are italicised had their means multiplied by − 1 as these studies measured negative effects but as a percentage (an increase in percentage is a negative outcome) and therefore were brought in line with the other studies. Abbreviations: *HRE*, hexanucleotide repeat expansion; *GFP*, green fluorescent protein; *DPR*, dipeptide repeat protein; *NMJ*, neuromuscular junction; *iPSCs*, induced pluripotent stem cells

## Results

**Fig. 2:**
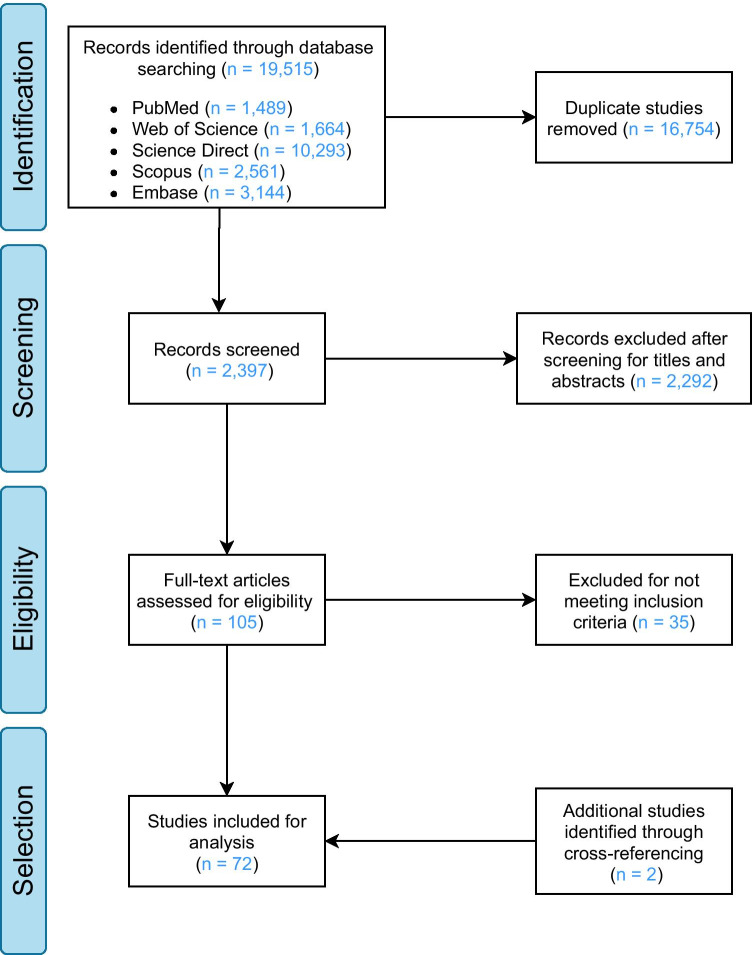
Inclusion of articles by Preferred Reporting Items for Systematic Reviews and Meta-Analyses (PRISMA) flow diagram

The searches conducted in PubMed, Scopus, Web of Science (WoS), EMBASE and Science Direct electronic databases yielded 1489, 2561, 1664, 3144 and 10,293 articles, respectively, reaching a total of 19,515 publications, of which 16,754 were identified as duplicates and removed from the data set. The titles and abstracts of the remaining 2397 articles were screened for eligibility, with 2292 publications deemed to fall outside the scope of the systematic review and excluded. Full texts of the final 105 articles were retrieved, read in full and carefully assessed against the inclusion criteria, with 70 studies deemed eligible for inclusion in the systematic review. Additionally, two relevant studies have been identified through cross-reference screening of relevant literature, giving rise to a total of 72 studies included in our analysis (see Fig. 2).

### General Characteristics of Selected Studies

**Fig. 3:**
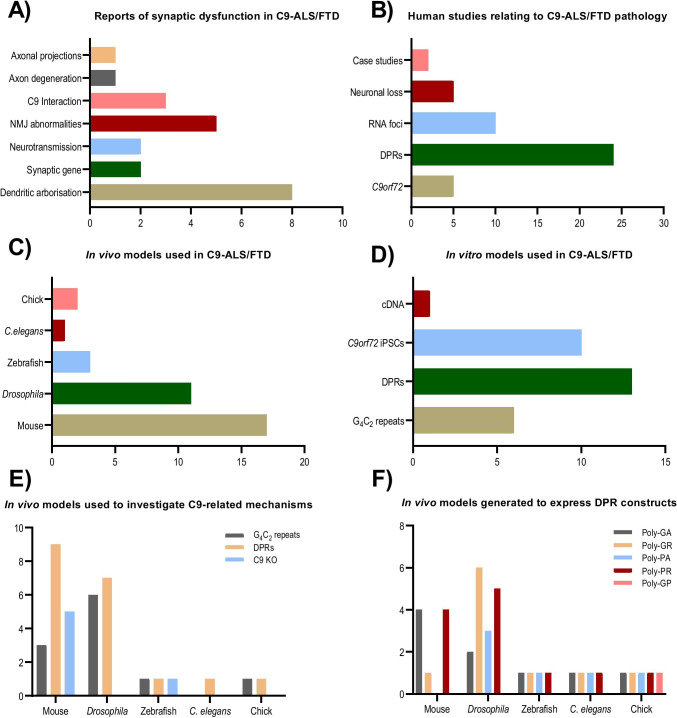
Frequency graphs illustrating the characteristics of selected studies. (**A**) Shows the number of studies that report synaptic dysfunction. (**B**) Illustrates the number of studies which report ALS/FTD pathology in C9-patients. (**C**) Shows the number of different animal models used in in vivo experiments. (**D**) Shows the number of different in vitro models used in the selected studies. (**E**) illustrates the pathomechanism type assessed in different animal models. (**F**) Illustrates the number of DPR constructs introduced to different animal models. Abbreviations: *DPRs*, dipeptide repeat proteins; *NMJ*, neuromuscular junction; *cDNA*, circular DNA

The studies selected for inclusion in the systematic review were published between 2011 and 2021 (*n* = 72). Of the papers included, approximately half used *C9orf72* mutation-positive subjects (*n* = 49) whilst the remaining papers attempted to recapitulate C9-ALS/FTD pathology in either in vitro models (*n* = 28) or in vivo models (*n* = 30). The characteristics of all selected studies, including the methodology and main findings reported, are summarised in Tables 2, 3 and 4 and in Fig. 3.

**Table 2:** Publications retrieved concerning synaptic function and morphology

Study	Methodology	Main findings
Bieniek et al. [107]	FTD-*C9orf72* and -progranulin patient tissue PCR, Thioflavin-S staining, IHC and IF	*• C9orf72* patients had higher degrees of neurofibrillary tangles and tau pathology when compared to other genetic causes of FTD *•* Sporadic cases of FTD had a similar Tau burden to *C9orf72* *•* P62 labelled neurofibrillary tangles and ubiquitin. Ubiquilin 2 was more specific in its labelling and labelled dystrophic neurites in the molecular layer of the dentate fascia and CA3 of the hippocampus
Devlin et al. [41]	Patient-derived iPSC MNs with *C9orf72* or *TARDBP* mutations RNA FISH, IF, repeat PCR, electrophysiology and cell viability assays	*•* Regardless of mutation, neurons were hyperexcitable followed by loss of action potential and synapse activity *•* Functional loss was a result of reductions in Na^+^ and K^+^ voltage currents *•* Despite loss in action potential output, neurons remained viable
Freibaum et al. [42]	Transgenic *Drosophila* lines expressing × 8, × 28 or × 58 G_4_C_2_ repeat-containing constructs; iPSCs-derived neurons generated from *C9orf72* patients; HeLa and HEK293T cells expressing (G_4_C_2_)_n_ repeats *Drosophila* phenotype analysis; IF; visualization of DPRs in *Drosophila*; immunoblots; RNA FISH	*•* Repeat length-dependent neurodegeneration accompanied by DPRs was demonstrated to occur in the transgenic *Drosophila* model *•* A large-scale unbiased genetic screen conducted in these animals identified 18 genetic modifiers encoding components of the nuclear pore complex (NPC) and nucleocytoplasmic transport *•* Defects in the morphology of nuclear envelope and defective RNA export were observed in cells lines expressing G_4_C_2_ repeats in vitro and in vivo *•* Changes in the NMJ were seen with reductions in synaptic boutons and active zones
Frick et al*.* [16]	Post-mortem tissue from ALS/FTD/ALS-FTD patients with *C9orf72* mutation and C9 negative controls; human iPSC derived motor neurons; C57BL/6 N and *C9orf72* KO mice Generation and characterisation of novel monoclonal antibodies against C9orf72; ICC, IF and biochemistry techniques to detect, quantify and co-localise C9 protein	*•* 80% reduction in the abundance of C9orf72 protein in the cerebellum of *C9orf72* mutation carriers compared to controls *•* No association between cerebellar levels of C9orf72 protein and clinical phenotypes, age of onset nor disease duration *•* Using novel monoclonal antibodies against C9orf72 shown C9 protein to be expressed presynaptically and interact with RAB3 proteins *•* Findings suggest C9 regulates synaptic vesicular functions and hence serves a physiological function in the brain
Hao et al. [56]	Transgenic mouse model expressing poly-PR under the control of neuronal *Thy1* promoter (GFP-PR_28_) Histopathological, behavioural, RNA sequencing and gene ontology analysis	*•* Motor deficits and ataxia-like phenotype in GFP-PR_28_ heterozygous mice *•* Cerebellar cortex atrophy *•* Loss of Purkinje cells *•* Increased microglial and astrocyte activation in the cerebellum and spinal cord *•* Dysregulation of synaptic transmission- and ER stress-related genes revealed by gene ontology
Herranz-Martin et al*.* [111]	Two mouse lines overexpressing either 10 pure or 102 interrupted G_4_C_2_ repeats mediated by AAV viral injection (i.e. HRE-10 and HRE-102 mice) Mouse behavioural testing, RNA FISH to detect intracellular RNA foci, IF, immunoblotting	*•* Purkinje cell layer of the cerebellum was one of the brain regions with the highest abundance of RNA foci per area, with no differences reported between *•* HRE-10 and HRE-102 mice brains at 12 months Poly-GA aggregates were widespread in the cerebellum of HRE-102 but not HRE-10 mice, accompanied by high expression of p62 *•* Despite an increase in markers of apoptotic cell death in HRE-102 animals, there was no evidence of cerebellar astrogliosis or neurodegenerative damage in HRE-102 mice *•* Infrequent TDP-43 aggregates were reported in the cerebellum of those animals, the majority of which were cytoplasmic *•* Synaptic pathology at NMJ
Jensen et al. [4]	20-month old transgenic mouse line expressing poly-GA_149_; Transfected primary rat cortical and motor neurons with poly-GA; Patient-derived *C9orf72* iPSC cells Immunocytochemistry, immunoblotting and IHC; live-cell imaging, qRT-PCR	*•* GA aggregates are mobile within the cytoplasm and axons of MNs and cortical neurons *•* Increased Ca^2+^ depolarisation but impaired synaptic unloading reduced synaptic function *•* Synaptic vesicle-associated protein 2 (SV2) was reduced in all models used in this study *•* Upon the introduction of exogenous SV2 in the mouse model, neuronal death was prevented
LaClair et al*.* [81]	Double transgenic mouse lines (poly-GA-Nes and -PR-Nes) generated by crossing poly-GA_175_ and GFP-PR_175_ mice (expressing poly-GA and poly-PR) with Nestin-Cre driver line to allow for CNS-wide neuronal high-level expression of the two key DPRs Quantitative ICC and IF of DPRs and NeuN-positive cells; Western blotting, ELISA, qPCR and gene ontology	*•* In vivo comparison of poly-GA and poly-PR toxicity revealed poly-GA to be the dominant driver of C9orf72-ALS/FTD pathogenesis with widespread expression of poly-GA, but not poly-PR, giving rise to disease-relevant phenotypes in the CNS in vivo *•* Poly-GA expression induced interferon responses resulting in inflammation, selective neuronal loss of spinal cord motor neurons, muscle denervation, TDP-43 inclusions in the forebrain and downregulation of synaptic genes, requiring euthanasia at 7 weeks of age
Lee et al. [39]	HEK-293 cells transfected with vectors containing 125 repeats of DPRs; chick embryos expressing DPR constructs; chick embryos electroporated with G_4_C_2_ constructs (8x, 38x, 72 × and 128); post-mortem tissue from *C9orf72* positive ALS cases IF staining; FISH; Western blotting; filter trap assay; TUNEL toxicity assay	*•* G_4_C_2_ repeats induce neurotoxicity in a length-dependent manner in vivo, with chick embryos electroporated with 38 × constructs showing the highest levels of cell death (TUNEL-positive cells) *•* RNA foci were abundant in the spinal cord of chicks electroporated with G_4_C_2_ repeats of disease-associated lengths (× 38, 72x, 128x). The highest prevalence of RNA foci was found in embryos expressing × 38 repeat constructs *•* Chick embryos expressing longer (> × 8) G_4_C_2_ constructs showed loss of motor neurons on the electroporated side of the spinal cord and motor axon pathway abnormalities with evidence of nerve truncation and failure of axon bundles to reach the periphery *•* Poly-GA was the most abundant DPR in the cortex of C9-ALS cases. It was also shown to be the most toxic in vitro and in vivo *•* DPRs were found to interact with each other, with poly-GA shown to sequester -GP and -PA when co-expressed in vitro. Dual expression of -GA and -PA ameliorated -GA-induced toxicity by inhibiting its aggregation in vitro and in vivo
Mackenzie et al. [26]	*C9orf72* patient brain tissue IHC, IF and haematoxylin and Eosin staining, immunoblotting, filter trap assays	*•* Poly-DPR inclusions were most common in order of GA > GP > GR > PR/PA in the frontal cortex and cerebellum *•* Moderate association of poly-GA positive dystrophic neurites and neurodegeneration in the frontal cortex *•* Total poly-GA burden correlated with disease onset
Maor-Nof et al. [82]	Wild-type and p53-knockout Mouse primary cortical neurons were transduced with poly-PR_50_; Poly-PR_50_ mouse model; (G_4_C_2_)_30_ expressing *Drosophila* line IF, fly-eye degeneration assay, survival assay, comet assay, immunoblotting, ATAC-sequencing, RNA-sequencing, CRISPR-Cas9	*•* Neurons expressing poly-PR and -GR activate p53 signalling, and reduction of p53 was sufficient to stop poly-PR and -GR mediated toxicity and increased the lifespan of a mouse model *•* P53 reduction rescued axonal degeneration elicited by poly-PR and -GR *•* P53 drives neurodegeneration by activation of *Puma*
May et al*.* [22]	Primary neuronal cell culture and HEK293 in vitro cultures transfected with constructs encoding synthetic genes for DPRs in the absence of G_4_C_2_ repeats; post-mortem tissue from *C9orf72* patients Analysis of DPR toxicity and aggregation properties; quantitative mass spectrometry to identify co-aggregates of Poly-GA	*•* P62 co-localises with DPRs, specifically poly-GA which sequesters Unc119 *•* Abundant poly-GA pathology in the cerebellum *•* P62-positive poly-GA aggregates, as well as knockdown of Unc119, inhibit dendritic arborisation and induce apoptotic cell death in vitro *•* Overexpression of Unc119 reduced poly-GA toxicity *•* In C9-patients, 1.6% of poly-GA inclusions in the cerebellum are Unc119-positive, compared to 9.5% of poly-GA inclusions in the frontal cortex
O’Rourke et al. [99]	Transgenic mice carrying bacterial artificial chromosome (BAC) containing human *C9orf72* gene with either the healthy allele (15 repeats) or 100–1000 repeats expansion — C9-BAC_exp_; primary cortical cultures generated from those mice FISH of sense and antisense RNA foci; immunostaining of DPRs; behavioural testing; histological examination of neuronal and motor damage; RNA-seq analysis	*•* Sense and antisense RNA foci were present throughout the CNS, including Purkinje cells and to a lesser degree cerebellar granular layer of C9-BAC_exp_ mice from F112 to F113 lines *•* Both soluble and insoluble fractions of poly-GP were abundant in the brains of C9-BAC_exp_ mice as young as 6 months *•* Despite the presence of DPRs and RNA foci, characteristic of C9-ALS/FTD pathology, the mice did not display any behavioural abnormalities nor neurodegeneration *•* There was no evidence of TDP-43, ubiquitin or p62 inclusions, and no signs of gliosis or inflammation *•* Neuronal loss was not observed, and synapses appeared to be unaffected *•* RNA-binding proteins Pur-α, hnRNPA3, hnRNPA2/B1 and hnRNP-H were not consistently co-localising with RNA foci in C9-BAC_exp_ mice, and there was no evidence of sequestration *•* Nucleolar integrity appeared to be disrupted in C9-BAC_exp_ mice carrying disease-associated repeat expansion *•* Dispersion of nucleolin from the nucleus was observed in those mice *•* Administration of antisense oligonucleotides targeting exon 2 of human C9orf72 suppressed RNA foci and DPRs in C9-BAC_exp_ mice primary cortical cultures
Park et al*.* [24]	*C9orf72 Drosophila* model — transgene lines expressing poly-PA, poly-GA, poly-PR and poly-GR Analysis of dendritic length and branching points of class IV dendritic arborisation (C4 da) neurons, RT-PCR analysis of CrebA mRNA levels	*•* Expression of arginine-rich DPRs (poly-PR and -GR) was associated with the most significant reduction in dendritic branches and number of Golgi outposts in dendrites of C4 da neurons *•* mRNA levels of CrebA transcription factor were markedly reduced in brains of *Drosophila* expressing poly-PR but not poly-GR
Perry et al*.* [44]	*Drosophila* lines for G_4_C_2_ repeats, poly-GR and SOD1 mutations IF, electrophysiology and behavioural assays	*•* Reductions in synaptic arborisation and active zones at the NMJ in third-instar larvae following G_4_C_2_ repeat transduction *•* Neurotransmission was reduced, but homeostatic plasticity of the junction was retained *•* Enhancing this plasticity can strengthen synaptic function even with C9 repeats being present
Saberi et al*.* [96]	Post-mortem brain and spinal cord tissue of C9-ALS patients Quantitative analysis of DPRs, nuclear pore proteins and C9orf72 protein	*•* Reduced levels of C9orf72 protein in frontal and occipital cortices compared to controls, with no change in the cerebellum *•* DPRs abundantly observed in granular, molecular and Purkinje cell layers of the cerebellum *•* Poly-GR DPRs abundantly localised in dendrites forming aggregates in the motor cortex *•* No DPR aggregates in axons nor axonal degeneration
Sellier et al. [18]	Mouse cortical neurons were transduced with shRNA against *C9orf72*; zebrafish with decreased C9orf72 and Ataxin2 mutant expression were generated via antisense oligonucleotides IF, immunoprecipitation, Western blot, novel antibody manufacture, behavioural studies, gross morphological analysis	*•* C9orf72 forms a complex with SMCR8 and WDR41 with subsequent interaction with RAB8a and RAB39b as a GTP/GDP exchange factor *•* Loss of C9orf72 does not impact cell viability but increases aggregation of TDP-43 and p62 *• C9orf72* is particularly deleterious in combination with *ataxin-2* Q30
Schweizer Burguete et al. [23]	(G_4_C_2_)_48_ repeat transduced *drosophila*; primary rat spinal cord neurons; *C9orf72* patient-derived iPSCs Live imaging, FISH, immunofluorescence	*•* RNA foci were localised to the nucleus and the cytoplasm and neurites *•* Neuritic foci alone correlate with neuronal branching deficits *•* RNA foci can be translocated across the neuron and knockout of transport systems such as Fragile X mental retardation protein (FMRP) prevents neuritic localisation and branching defects
Swaminathan et al. [74]	Zebrafish model transiently expressing constructs containing DPRs of varying lengths (40, 200 and 1000); transgenic zebrafish line expressing 100 repeats of poly-GR Touch-evoked escape response test; Western blotting; acridine orange staining; assessment of motor neuron morphology	*•* Expression of poly-GR was associated with the greatest incidence of developmental lethality and morphological defects in zebrafish. Poly-GA was found to be the least toxic out of the five DPRs studied *•* Expression of 1000 repeats of any of the DPRs, even the ‘non-toxic’ poly-GA induced locomotor deficits in zebrafish *•* Poly-GR affected motor neuron growth in transgenic zebrafish line overexpressing 100 -GR repeats
Xiao et al. [17]	*C9orf72* knockout mice and Wild-type tissues Immunoprecipitation, electrophoresis and immunoblotting, IF, synaptosome fragmentation	*•* C9orf72 is localised to both the presynaptic and postsynaptic compartments *•* Despite *C9orf72* knockout, expression levels of presynaptic compartments did not vary in the forebrain *•* Post-synaptic compartments showed loss of Smcr8 protein, reductions in Rab39b and upregulated GluR1. This change in expression was visualised in the dorsal hippocampus
Xu and Xu [21]	UAS-DPR *Drosophila* were generated expressing poly-PR_36_, -GR_36,_ and -PA_36_ Behavioural assays and lifespan assay; immunoblotting and IHC, live imaging, qPCR, drug treatment	*•* Glutamatergic neuronal degeneration is observed upon transfection in *Drosophila* *•* Poly-GR and -PR expressing neurons had higher levels of intracellular Ca^2+^ *•* Increased synaptic boutons and active zones in larval NMJs *•* Arginine DPR-dependent NMDA-dependent excitotoxic mechanisms were reported in the presynaptic terminal of glutamatergic neurons
Yang et al. [77]	Transgenic *Drosophila* lines expressing poly-GR_80_ and -PR_80_ constructs; iPSC-derived cortical neurons generated from *C9orf72* patients; post-mortem brain tissue of C9 cases; HeLa cells transfected with (GR)_80_ Fly wing notching phenotyping; climbing assay and quantification of dendritic branching; IHC, qRT-PCR	*•* Expression of poly-GR_80_ and -PR_80_ constructs induce toxicity in both neuronal and non-neuronal cells of *Drosophila* *•* Poly-GR_80_ is present predominantly in the cytosol with negligible expression in the nucleolus and results in suppression of Notch signalling and loss of cells in the wings of those animals and decreased dendritic branching in sensory neurons *•* iPSC-derived cortical neurons generated from *C9orf72* patients and post-mortem brain tissue of C9 cases show a downregulation of Notch target genes *•* Co-expression of poly-GR_80_ and -PR_80_ resulted in sequestration of -GR by -GP which ameliorated -GR toxicity in vitro and restored Notch signalling in *Drosophila*
Zhang et al*.* [28]	Cultured cells and primary neurons transfected with GFP-GA_50_, GFP-GP_47_, GFP-GR_50_, GFP-PR_50_ or GFP-PA_50_ expression vectors; post-mortem tissue from C9-ALS/FTD cases IHC; electron microscopy; immune-electron microscopy; FISH; live cell imaging; Western blot; qRT-PCR	*•* Expression of poly-GA induces formation of soluble and insoluble species and filament-rich inclusions in vitro and in vivo *•* Poly-GA activates caspase-3 apoptotic pathway and leads to neurite outgrowth impairment, inhibition of proteasomal activity and ER stress *•* Administration of ER inhibitors protected against poly-GA-induced toxicity

Abbreviations: *AAV*, adeno-associated virus; *ALS*, amyotrophic lateral sclerosis; *ATAC*, assay for transposase-accessible chromatin; *Ca*^*2*+^, calcium ions; *CA3*, Cornu Ammonis 3; *Cas9*, CRISPR-associated protein 9; *CNS*, central nervous system; *CrebA*, cyclic AMP response element binding protein A; *CRISPR*, clustered regularly interspaces short palindromic repeats; *DPRs*, dipeptide repeats; *ELISA*, enzyme-linked immunosorbent assay; *ER*, endoplasmic reticulum; *FISH*, fluorescent in-situ hybridisation; *FTD*, frontotemporal dementia; *GDP*, guanosine diphosphate; *GFP*, green fluorescent protein; *GluR1*, glutamate receptor 1; *GTP*, guanosine triphosphate; *HEK293T*, human embryonic kidney cells; *hnRNP*, heterogenous nuclear ribonucleoproteins; *HRE*, hexanucleotide repeat expansions; *ICC*, immunocytochemistry; *IF*, immunofluorescence; *IHC*, immunohistochemistry; *iPSC*, induced pluripotent stem cells; *K*^+^, potassium ions; *KO*, knockout; *MNs*, motor neurons; *Na*^+^, sodium ions; *NeuN*, neuronal nuclei; *NMJ*, neuromuscular junction; *p53*, tumour protein 53; *p62*, ubiquitin-binding protein; *PCR*, polymerase chain reaction; *PUMA*, p53 upregulated modulator of apoptosis; *Pur-α*, purine-rich element binding protein; *qRT-PCR*, quantitative real-time PCR; *RAB*, ras-associated binding; *RNA*, ribonucleic acid; *shRNA*, small hairpin RNA; *SMCR8*, Smith-Magenis syndrome chromosomal region candidate gene 8; *SOD1*, superoxide dismutase 1; *TARDBP*, Tar-DNA binding protein; *TDP*-43, Tar-DNA binding protein 43; *Thy1*, thymocyte differentiation antigen 1; *TUNEL*, terminal deoxynucleotidyl transferase dUTP nick end labelling; *UAS*, upstream activation sequence; *WDR41*, WD repeat domain 41

**Table 3:** Publications retrieved concerning findings in the cerebellum

Study	Methodology	Main findings
Al-Sarraj et al*.* [25]	Post-mortem tissue from MND/ALS, FTLD and ALS-FTLD cases with, and FTLD cases without C9 expansion mutation Neuropathological assessment, IHC and IF studies	*•* p62 positive, p-TDP-43 negative inclusions were reported in cerebellar Purkinje cells and molecular layer of C9-positive cases
Ash et al. [10]	Human *C9orf72* tissue Meso scale discovery assay, IHC, IF; generation of novel antibodies to poly-GA, GP and -GR	*•* C9orf72 RAN translation products (now called DPRs) were detected in high molecular weight aggregates specific to *C9orf72* expansion and not other neurodegenerative or CAG expansion disorders *•* DPRs were abundant in the cerebellum of *C9orf72* cases
Belzil et al. [103]	*C9orf72* patient tissue, skin biopsies and blood samples with derivation of fibroblasts qRT-PCR, ddPCR and chromatin immunoprecipitation	*•* Both the frontal cortices and cerebellum had reductions in *C9orf72* mRNA in pathogenic expansions *• C9orf72* with expansions increases the rate of binding to trimethylated lysine residues within histones H3 (H3K9/K27/K79) and H4 (H4K20) which is detectable in the blood of patients
Chew et al*.* [94]	Transgenic mouse model expressing (G_4_C_2_)_66_ throughout the CNS by means of AAV-mediated somatic brain transgenesis Behavioural testing; RNA FISH detection of RNA foci, immunoassays for DPRs	*•* RNA foci were abundant in Purkinje cells of (G_4_C_2_)_66_ mice at 6 months, with 40–54% of Purkinje cells foci-positive, and to a lesser degree, in granular and molecular layers of cerebellum *•* Poly-GA, poly-GP and poly-GR inclusions were detected in the cerebellum, accompanied by a loss (11% decrease) of Purkinje cells in (G_4_C_2_)_66_ mice *•* A decrease in overall brain weight, indicative of repeat expansion-mediated atrophy, and behavioural/ motor skill abnormalities were also reported
Cooper-Knock et al*.* [109]	Samples from ALS/FTD cases with and without C9 expansion mutation, healthy controls and asymptomatic C9 carriers Identification of nuclear and cytoplasmic RNA foci using FISH, investigation of RNA foci binding partners using mass spectrometry, pulldown assays and IHC	*•* RNA foci were found to be abundant in the cerebellar granule cells of C9-ALS/FTD patients and absent in cases without C9 mutation and healthy controls (although the levels varied based on clinical presentation) *•* A number of putative binding partners of RNA foci were identified, including hnRNP, which was shown to co-localise with RNA foci in cerebellar granule cells
Cooper-Knock et al. [112]	*C9orf72* patient tissue IHC, FISH, RNA-binding assays	*•* Antisense RNA foci are predominantly present in the cerebellar Purkinje cells and motor neurons. In motor neurons, antisense foci (and not sense) correlated with TDP-43 pathology *•* Sense RNA foci localised predominantly to the granular cells of the cerebellum *•* DPRs were present with the greatest frequency in granular cells of the cerebellum and then the motor neurons of the spinal cord *•* Antisense RNA foci co-localised with SRSF2, hnRNP A1, hnRNP A/F, ALYREF and hnRNP K
Corcia et al*.* [60]	*C9orf72* mutation carrier presenting with pure cerebellar syndrome Case study report	*•* Female with family history of ALS and other neurodegenerative disorders presents with symptoms of pure cerebellar syndrome — locomotor disturbance, cerebellar vermis atrophy and no cognitive dysfunction, and is subsequently found to be a carrier of *C9orf72* mutation *•* Demonstrating a link between C9 mutation and cerebellar pathology
Davidson et al. [89]	Post-mortem tissue from FTLD-tau, FTLD-TDP type A, B and C, and *C9orf72* expansion, *MAPT* or *GRN* mutation carriers, and healthy controls IHC staining and pathological assessment	*•* Investigated the patter of hnRNP A1, A2/B1 and A3 staining across brain regions in various subtypes of FTLD cases, including carriers of *C9orf72* mutation *•* No difference in the intensity and staining pattern of hnRNP A1 or hnRNP A2/B1 was observed in C9orf72-positive cases compared to other FTLD cases, with no hnRNP A1 or A2/B1 immunoreactive inclusions reported *•* hnRNP A3-positive inclusions were seen in cerebellum of some C9orf72 cases
Davidson et al. [105]	*C9orf72* patient tissue and other disorders (Huntington’s/FTD-TDP and Alzheimer’s) IHC, antibody comparison	*•* Confirming previous studies, C9-L antibodies labelling diffuse cytoplasmic staining and speckles in the Purkinje neurons of the cerebellum *•* Additionally, C9-S labelled the nuclear membrane only *•* ProteinTech’s monoclonal and polyclonal antibody as well as GeneTex achieved similar staining to that of C9-L *•* Nuclear membrane staining of C9-S was shifted to the plasma membrane in the spinal cord of sufferers *•* Commercial antibodies were unable to recapitulate C9-S staining
DeJesus-Hernandez et al*.* [37]	Cerebellum and frontal cortex tissue of C9 expansion mutation carriers Detection and visualisation of RNA foci by RNA FISH and IF staining followed by computer-assisted quantification and co-localisation	*•* RNA foci were abundant in the cerebellum of C9 carriers, with 23% and 1% of granule cells containing sense and antisense RNA foci, respectively *•* RNA foci were most abundantly present in cerebellar Purkinje cells, with approximately 70% of all cells containing RNA foci *•* Increased percentage of Purkinje cells containing antisense RNA foci was associated with delayed age at disease onset
Fogel et al*.* [59]	Adult-onset sporadic ataxia cases Ataxia patients were screened for common genetic spinocerebellar ataxias and tested for *C9orf72* expansion mutation	*•* The majority of patients were diagnosed as having pure cerebellar ataxia *•* Out of the 209 ataxia patients tested for C9 mutation, only one positive case was identified *•* The C9-positive ataxia patient has shown no motor neuron deterioration or cognitive dysfunction
Gendron et al*.* [27]	Post-mortem tissue from ALS, FTLD, MND and neurodegenerative disease with and without C9 mutation; HeLa cells and HEK293T cells transfected with either (C_4_G_2_)_66_ or (C_4_G_2_)_2_ expression vectors Generation of novel antibodies to visualise C_4_G_2_ RAN proteins – poly-PR, -GP and -PA, FISH to detect RNA foci formed from antisense transcripts in cultured cells, FISH detection of RNA foci and IF staining of human post-mortem tissue	*•* Ectopic expression of (C_4_G_2_)_66_ leads to formation of RNA foci and synthesis of RAN proteins in cultured cells *•* (C_4_G_2_)_n_ RNA foci were detected in the cerebellum of C9-ALS/FTD patients, predominantly in the Purkinje cell layer, in both astrocytes and neurons *•* Poly-PA, poly-GP and poly-PR inclusions synthesised from antisense repeat were present in the brain of human C9-ALS/ FTD cases *•* The cerebellar granule cells of C9-ALS/FTD patients were shown to be particularly abundant in poly-GP, whereas poly-PA and poly-PR pathology was markedly less pronounced
Gendron et al*.* [29]	Post-mortem tissue from *C9orf72* mutation carriers with a diagnosis of ALS, FTLD and FTLD-MND Quantitative immunoassays; IHC; analysis of patients’ clinical data	*•* Poly-GP levels were highest in the cerebellum of *C9orf72* cases *•* Cerebellar poly-GP load was markedly lower in patients with ALS as compared to those with FTLD or FTLD-MND *•* There was an association between cerebellar poly-GP levels and cognitive scores in C9-ALS cases
Goldman et al*.* [58]	Family with multiple system atrophy (MSA) and ALS, positive for C9 expansion mutation Case study report	*•* Carriers of *C9orf72* expansion mutation can present with both MSA and ALS, highlighting the possibility of large phenotypic variability associated with the C9 mutation *•* Draws a link between C9 mutation, ALS and cerebellar ataxia
Lee et al*.* [36]	(G_4_C_2_)_n_ transfected human non-neuronal cell lines and rat primary cortical neurons; zebrafish embryos injected with EGFP constructs containing 8x, 38 × and 72 × G_4_C_2_ repeats; human cerebellum tissue from ALS and FTD cases with confirmed C9 expansion mutation Detection of RNA foci by FISH, immunoprecipitation, FACS and ICC analysis for expression of apoptosis markers, detection and co-localisation of DPRs	*•* RNA containing 38 × and 72 × G_4_C_2_ repeats caused cellular toxicity in a length-dependent manner in transfect cell lines and in vivo zebrafish model *•* G_4_C_2_ foci induced apoptotic cell death, resulting in loss of foci-positive cells and increased expression of Annexin V and Caspase-3 apoptotic markers *•* RNA foci were found to be abundant in the cerebellum of ALS/FTD cases with C9 expansion mutation *•* Intranuclear neuronal RNA foci were larger (~ 500 nm) in the cerebellum tissue of C9-ALS/FTD than cortex (~ 200 nm), with over 70% of cerebellar foci co-localised with hnRNP-A3 *•* Co-localisation of poly-GA with poly-GR and -PR was reported to occur infrequently *•* Poly-GA was capable of sequestering poly-GP and -PA
Mackenzie et al. [30]	Cohort of 35 cases with a broad spectrum of clinical phenotypes, positive for *C9orf72* mutation Characterised novel monoclonal antibodies against poly-GA; immunoblotting	*•* The pattern of DPR expression was similar in all cases, regardless of the diagnosis *•* Highest abundance of DPRs was found in the cerebellum, neocortex and hippocampus *•* There was no correlation between DPR pathology burden and the severity of neurodegeneration
Mahoney et al. [80]	Cases with *C9orf72* mutation and syndromic diagnosis within FTLD spectrum Clinical, histopathological and neuroimaging analysis of C9orf72 expansion mutation	*•* Large clinical heterogeneity was observed among *C9orf72* patients *•* Anxiety and memory impairments were commonly reported *•* Extensive thinning of frontal, parietal, occipital lobes and cerebellar atrophy was observed *•* Abundant expression of p62 inclusions was seen in the hippocampus and cerebellum
Mann et al. [31]	Post-mortem tissue from *C9orf72* mutation-positive cases with confirmed diagnosis of FTLD or MND and p62-positive inclusions IHC; southern blotting	*•* DPRs were shown to be major components of p62-positive inclusions in the cerebellum and hippocampus of C9-FTLD and MND cases *•* There was some evidence of antisense translated DPRs; however, the poly-AP staining was variable
Mizielinska et al*.* [108]	Post-mortem tissue from C9-FTLD cases, neurodegenerative disease and healthy controls FISH and protein immuno-staining to detect, quantify and determine the subcellular localisation of sense and antisense RNA foci	*•* The presence of both sense and antisense RNA foci was reported in the brains of C9-FTLD cases including the cerebellum *•* RNA foci occurred more frequently in neurons than glia *•* RNA foci were seen in TDP-43 and p62-positive neurons (which were particularly abundant in the cerebellum), but the frequency was not greater than would be expected to occur by chance
Mori et al. [85]	Post-mortem tissue from *C9orf72*-positive ALS/FTD cases, patients with other neurodegenerative diseases and healthy controls Generation of novel antibodies against antisense-translated DPRs and putative carboxy-terminal tails of poly-GP, -GR and -GA reading frames; IHC analysis; ELISA and immunoblotting	*•* Demonstrated that G_4_C_2_ repeat is bidirectionally translated into co-aggregating DPRs in patients carrying *C9orf72* expansion mutation *•* Non-ATG translation extends past the G_4_C_2_ repeat region in *C9orf72* patients, as demonstrated using novel antibodies raised specifically against the putative carboxy-terminal tail of DPRs in poly-GA, -GP and -GR reading frames *•* The pattern of poly-GR inclusion pathology was shown to follow a rostro-causal gradient, with neuronal cytoplasmic inclusions (NCIs) abundantly found in the molecular and granular layer of the cerebellum, but rarely in Purkinje cells
Quaegebeur et al. [91]	Brain homogenates of FTD patients with *C9orf72* expansion mutation Meso scale discovery (MSD) immunoassay	*•* The highest abundance of DPRs was detected in the cerebellum of C9-FTD cases *•* Relative DPR solubility was highest in the cerebellum *•* Levels of poly-GR and relative solubility of poly-GP were correlated with clinical parameters
Renton et al*.* [2]	ALS-FTD cases with a positive linkage to the chromosome 9p21 region Next-generation sequencing of chromosome 9p21 region; FISH analysis; expression arrays; RT-PCR	*•* Identified GGGGCCC hexanucleotide repeat expansion in *C9orf72* gene as the cause of 9p21-linked ALS-FTD disease *•* Highest expression levels of *C9orf72* RNA were detected in the cerebellum
Schludi et al. [88]	Transgenic mice expressing (poly-GA)_149_ conjugated with cyan fluorescent protein (CFP) under the control of Thy1 promotor — Thy1 (GA)_149_-CFP mice; post-mortem *C9orf72*-ALS/FTD patient samples; primary hippocampal neurons from rats transduced with lentiviral vector containing (GA)_175_-GFP cDNA IHC and IF staining, immunoassays; qRT-PCR; behavioural and clinical assessment of mouse motor function	*•* Poly-GA aggregates were abundant in the spinal cord and brainstem of (GA)_149_-CFP mice at 4–6 months of age. Poly-GA inclusions were also detected in the cerebellar nuclei but not the molecular or granular layer of the cerebellum of those animals *•* The majority of poly-GA inclusions were p62-positive and frequently co-localised with Rad23b *•* There was no evidence of Unc119 sequestration and no nucleolar pathology *•* M1f2 (chaperone-associated protein) was shown to co-aggregate with a large proportion of poly-GA inclusions in the spinal cord of (GA)_149_-CFP mice, which was not the case in human *C9orf72* patients *•* Motor behaviour deficits were observed in (GA)_149_-CFP mice including balance and gait and decreased locomotor activity *•* Upregulation of neuroinflammatory markers was detected in (GA)_149_-CFP mice at 6 months *•* No neuronal loss and no signs of motor neuron degeneration were observed
Tan et al. [9]	ALS and FTLD cases mostly without the *C9orf72* expansion mutation, and healthy controls Cognitive, neuropsychiatric and functional assessment of patients; neuroimaging data	*•* Atrophy of cerebellar grey matter was observed across the spectrum of ALS-FTD *•* Correlation between neuropsychiatric function and atrophy of the crus and superior lobule of cerebellum was found *•* Motor symptoms were associated with atrophy of the inferior lobules
Tan et al*.* [70]	Post-mortem cerebellum tissue from *ATXN2*- and C9-ALS cases, sporadic ALS disease and sporadic muscular atrophy Histopathological analysis of cerebellar Purkinje and granule cell integrity	*•* Significant loss of Purkinje cells was observed in *ATXN2*-ALS *•* Despite a markedly higher abundance of TDP-43, p62- positive and poly-GA inclusions in the cerebellum of C9 mutation carriers compared to ATXN2-ALS cases, neuronal integrity appeared intact with no loss of Purkinje nor granule cells
Troakes et al. [20]	*C9orf72* patient tissue Case study, IHC, Western blot	*•* Star-shaped p62 inclusions in the cortex, basal ganglia and hippocampus *•* Few TDP-43 inclusions in the brain, pathology more abundant in the spinal cord such as other types of ALS *•* Cerebellum granular cells had p62-positive TDP-43 negative inclusions
van Blitterswijk et al*.* [[92]	Post-mortem cerebellar and/ or frontal cortex tissue from *C9orf72* expansion mutation carriers qRT-PCR and digital molecular barcoding techniques to assess total C9 transcript and variant (1–3) levels; immunoassay of DPRs	*•* A decrease in the abundance of total *C9orf72* transcript and variants 1 and 2 were detected in C9 carriers compared to controls *•* The strongest effect was seen in variant 2 — with qRT-PCR and digital barcoding showing 43% and 31% reduction in cerebellum, respectively *•* Intron-containing transcripts were associated with poly-GP and poly-GA levels in cerebellum of C9 carriers
Waite et al*.* [93]	Subjects with confirmed diagnosis of ALS, FTLD or ALS-FTD Southern blot detection of *C9orf72* expansion; qPCR analysis of C9 transcript levels; generation of polyclonal antibody against C9orf72; immunoblotting analysis of C9 protein level	*•* Southern blotting analysis of C9orf72 repeat expansion size revealed cerebellar tissue to have reduced modal expansion size compared to other brain regions *•* Expression of C9 transcript was significantly reduced in the cerebellum *•* Significant reduction in 48-kDa isoform of C9 protein was reported in the frontal cortex, but not in the cerebellum
Xiao et al*.* [104]	Post-mortem tissue from ALS cases with confirmed C9orf72 mutation, non-C9-ALS cases and healthy control Development of novel antibodies against C9-L and C9-S isoforms of C9orf72; investigation of the properties, abundance and subcellular localisation of C9 isoforms using IHC, Western blotting and immunoprecipitation	*•* No significant changes in the abundance of C9-L or C9-S were reported in the cerebellum of C9-ALS cases vs non-C9-ALS *•* Distinct subcellular localisation of the two isoforms was reported *•* C9-L isoform exhibited diffuse labelling in the cytoplasm of cerebellar Purkinje cells, with a striking labelling of numerous speckles observed in the neuronal perikarya and dendritic processes of both C9-carriers and non-carriers *•* C9-S antibody gave a very specific labelling of the nuclear membrane *•* C9 isoforms interacted with β1 and Ran‐GTPase components of the nuclear pore complex and thus were suggested to play a role in the disruption of the nucleocytoplasmic transport *•* No evidence of cerebellar neurodegeneration nor loss of Purkinje cells
Zhang et al. [65]	Transgenic mouse model expressing GFP-(GR)_100_ in the brain; transfected HEK293T cells expressing GFP-(GR)_100_ constructs; post-mortem cerebellar and cortical tissue from C9-ALS/FTD patients Mouse behavioural test; IHC and IF staining; Western blot; FISH; RNA-Seq and gene ontology; qPCR; RT-PCR; in vivo SUnSET assay	*•* GFP-(GR)_100_ mice accumulated diffuse, cytoplasmic poly-GRs which were associated with age-dependent neurodegeneration, brain atrophy, memory and locomotor deficits *•* Loss of cerebellar Purkinje cells was observed in GFP-(GR)_100_ mice *•* Poly-GR was found to co-localise with ribosomal subunits of eIF3η in GFP-(GR)_100_ mice and post-mortem tissue from C9-ALS/FTD patients *•* Poly-GR induced the formation of stress granules in transfected HEK293T cells
Zhang et al. [69]	Mouse model expressing poly-PR mediated through AAV1 viral injection of GFP-PR_50_; post-mortem frontal cortical tissue from ALS and FTD cases with C9 mutation; human iPSC-derived neurons Mouse behavioural testing, histopathological analysis, RNA, protein and IHC and IF analysis, gene ontology	*•* GFP-PR_50_ mice showed motor dysfunction and cognitive deficits, accompanied by reduced brain weight, age-dependent loss of poly-PR expressing Purkinje cells and cortical neurons at 3 months of age, suggestive of poly-PR-induced cell-autonomous neuron death *•* Increased astrogliosis and microgliosis in the cortex and cerebellum of GFP-PR_50_ animals were also reported *•* Poly-PR localised to heterochromatin, causing abnormal histone H3 methylation in mice and C9-ALS/FTD tissue

Abbreviations: *AAV*, adenovirus; *ALS*, amyotrophic lateral sclerosis; *ALYREF*, Aly/REF export factor; *ATXN2*, Ataxin-2; *ddPCR*, droplet digital PCR; *DPR*, dipeptide protein; *EGFP*, enhanced green fluorescent protein; *ELISA*, enzyme-linked immunosorbent assay; *FACS*, fluorescence-activated cell sorting; *FISH*, fluorescent in-situ hybridization; *FTD*, frontotemporal dementia; *FTLD*, frontotemporal lobar dementia; *GRN*, progranulin; *HEK293T*, human embryonic kidney 293 cells; *hnRNP*, heterogeneous nuclear ribonucleoproteins; *ICC*, immunocytochemistry; *IF*, immunofluorescence; *IHC*, immunohistochemistry; *iPSCs*, induced pluripotent stem cells; *MAPT*, microtubule-associated protein Tau; *MND*, motor neuron disease; *p62*, ubiquitin binding protein p62; *p-TDP-43*, phosphorylated TAR-DNA binding protein 43; *qRT-PCR*, quantitative real time PCR; *Rad23b*, UV excision repair protein homolog B; *RAN*, repeat-associated non-AUG; *RNA*, ribonucleic acid; *RNA-Seq*, RNA sequencing; *RT-PCR*, real time PCR; *SRSF2*, serine and arginine rich splicing factor 2; *SUnSET*, surface sensing of translation; *TDP-43*, TAR-DNA binding protein 43; *Unc119*, uncoordinated 119

**Table 4:** Publications retrieved concerning C9orf72-related pathology

Study	Methodology	Main findings
Atanasio et al. [97]	*C9orf72* haploinsufficiency model (*C9orf72*^−/−^) generated by replacing mouse *C9orf72* coding sequence and introns with lacZ reporter Behavioural and clinical examination of motor function; H&E and IHC analysis; RNA-seq	*• C9orf72*^−/−^ mice showed mild motor dysfunction including progressive muscle weakness observed at 40 weeks of age, tremor, rigidity and reduced locomotor behaviour *•* Enlargement of cervical lymph nodes and splenomegaly was observed, as well as inflammatory infiltrates in multiple organs *•* Serum levels of cytokines were elevated *•* Enrichment of immune-related transcripts, indicative of systemic immune response, was detected in *C9orf72*^−/−^ mice *• C9orf72*^−/−^ mice exhibited autoimmunity and age-related proliferative glomerulonephropathy
Darling et al. [78]	NSC34 cells transfected with DPR-containing plasmids; primary mouse neurons IF; Western blot; isothermal titration calorimetry (ITC); circular dichroism (CD) spectroscopy; fluorescence spectroscopy; dynamic light scattering (DLS); nanoparticle tracking analysis; transmission electron microscopy (TEM)	*•* Co-expression of DPRs results in altered cellular outcomes as compared to expression of single DPRs, suggesting that complex interaction occurring between individual DPRs can change their intrinsic properties and toxicity *•* Dual expression of poly-PR and -GA resulted in altered subcellular localization, morphology and amelioration of -PR-induced cytotoxicity
DeJesus-Hernandez et al. [1]	Patients with a *C9orf72* expansion mutation; post-mortem patient tissue PCR and qPCR, g/cDNA sequencing, western blotting, IHC and FISH	*•* First report of the *C9orf72* expansion as a cause of ALS/FTD *•* The most common genetic cause of FTD (11.7%) and ALS (22.5%) *•* Expansion in *C9orf72* led to nuclear foci formation in patients in the frontal cortex and spinal cord
Farg et al. [102]	Neuronal cell lines — murine neuro2a and human SH-SY5Y transfected with *C9orf72* siRNA; primary cortical neurons of C57B1/6 mice; post-mortem spinal cord tissue from *C9orf72* patients IF and immunoblotting to investigate subcellular localisation of C9orf72 protein; ICC, IHC and immunoprecipitation to detect co-localisation of C9 protein with Rabs; siRNA transfection of SH-SY5Y cells; transfection of neuro2a cell lines with C9orf72-GFP and LC3; mass spectroscopy to identify C9-interacting proteins	*•* Investigated cellular function and subcellular expression of C9orf72 protein *•* Evidence for C9orf72 involvement in intracellular trafficking and protein degradation *•* Demonstrated co-localisation of C9orf72 with Rab proteins — involved in autophagy and endosomal trafficking — in cell lines, mouse primary neurons and spinal cord of *C9orf72* patients *•* siRNA-induced depletion of C9 protein in transfected cells inhibited endocytosis *•* Ubiquilin-2, hnRNPA1 and hnRNPA2/B1 were shown to interact with endogenous C9orf72 in vitro *•* Inhibition of the proteasome promoted co-localization of C9orf72 and ubiqilin-2 in neuro2a cells treated with lactacystin *•* Inhibition of the proteasome in neuro2a cells transfected with C9orf72-GFP constructs induced the formation of stress granules and C9-aggregates
Koppers et al. [98]	Conditional *C9orf72* knockout mouse model — *C9orf72*^fl/fl^ mice crossed with Nestin-Cre mice to selectively ablate expression of *C9orf72* from neurons and glial cells IHC analysis of motor neurons and neuromuscular junction integrity, gliosis and TDP-43 inclusions; FISH, Western blotting; motor function testing	*•* Conditional knockout of *C9orf72* gene resulted in significant loss of body weight in mice but was not sufficient to reduce survival, induce neurodegeneration or affect motor function *•* No inflammatory responses or other pathological hallmarks of C9-ALS/FTD were detected in those mice
Lopez-Gonzalez et al. [84]	Transgenic *Drosophila* models including *Vg-Gal4*-GR_80,_ *UAS*-(G_4_C_2_)_58_, *GMR-Gal5*, *UAS*-(G_4_C_2_)_58_/*TM6B* and *Tb* lines; CRISPR-Cas9-edited iPSCs lines generated from *C9orf72* patients Genetic modifier screen; *Drosophila* eye phenotyping; ELISA; immunoblotting; generation of lentiviral particles expression Ku80 shRNA and sdRNA	*•* A genetic modifier screen using transgenic *Drosophila* as a model identified 19 genes whose partial loss of function suppressed poly-GR toxicity, one of which was Ku80, a key DNA repair protein *•* The levels of Ku80 expression were markedly elevated in poly-GR expressing flies and *C9orf72* iPSC-derived neurons *•* This was associated with increased levels of P53, phosphorylated ATM and apoptotic markers in C9orf72 patient neurons *•* Partial loss of function of Ku80 suppressed poly-GR-induced neuronal cell death in poly-GR expressing flies *•* CRISPR-Cas9-mediated deletion of G_4_C_2_ expansion repeats prevented elevation of Ku80 expression and downstream apoptotic markers *•* Small RNAs-mediated knockdown and CRISPR-Cas9-mediated ablation of Ku80 resulted in suppression of the apoptotic pathway
Mehta et al. [110]	*C9orf72* patient tissue BaseScope™ ISH	*•* BaseScope is a highly sensitive form of in situ hybridisation that improves signal and detection of RNA foci *•* Sense foci are associated with TDP-43 aggregation in spinal cord motor neurons but not spinal cord glia or indeed the motor cortical neurons *•* No correlation between TDP-43 and foci in areas outside of motor control was seen
Mizielinska et al. [64]	*Drosophila* generated to express DPRs under UAS promotor with 36 or 103 repeats; neuronal transfection with poly-GR_100_ and -PR_100_ Northern blotting, FISH, Immunoblotting, egg-adult viability, eye phenotyping, lifespan assay	*•* Gene expression of DPRs was switched on post-development in flies and caused fly death within 30 days. Reducing protein output of DPRs attenuated the lifespan reduction *•* Protein only poly-GR and -PR were compared to poly-GA and -PA with the former causing lethality and the latter having no effect on the fly *•* Neuron viability was reduced upon transfection with poly-GR_100_ and poly-PR_100_ *•* Poly-GA inclusion was a poor predictor of neurodegeneration
Mori et al*.* [85]	*C9orf72* patient tissue Filter trap assay; immunoblotting; RT-PCR; qPCR; IHC; IF	*•* Discovered that most of the TDP-43-negative inclusions characteristic of C9-ALS/FTD contain predominantly poly-GA proteins and to a lesser extent poly-GP and poly-GA
Mori *et* al. [59]	HEK293 cells transfected with plasmids containing 7, 17 or 23 repeats of G_4_C_2_; in vitro transcription of RNA probes; post-mortem tissue from *C9orf72* cases LC–MS identification and quantification of proteins; Western blotting; IF and IHC	*•* 20 RNA-binding proteins were identified as capable of binding to G_4_C_2_ repeats in vitro *•* Out of those, a few were selected to be further validated in post-mortem brain tissue of *C9orf72* cases including hnRNP A3 which was found to form neuronal cytoplasmic and intranuclear inclusions in the hippocampus *•* hnRNP A3-positive inclusions were of the p62-positive/TDP-43-negative type
Rudich et al. [72]	*C. elegans* model expressing 50 repeats of poly-GA, -PA, -GR or -PR DPRs Detection of DPRs by fluorescence microscopy; FRAP studies; paralysis and thrashing assays; brood size assays; neurodegeneration assays	*•* Expression of arginine-rich, but not alanine-rich DPRs induced toxicity in neuronal and non-neuronal contexts *•* This poly-PR and -GR-induced toxicity was dependent on the nuclear localization of the DPRs *•* The toxicity of -PR and -GR was found to be age dependent *•* Uncoupling of physiological aging from chronological aging ameliorated -PR but not -GR toxicity
Schludi et al. [32]	Transfected rat primary neuronal cultures expressing GA_175_, GR_149,_ GP_80_ or PR_175_; post-mortem brain and spinal cord tissue from *C9orf72* mutation patients IHC; RNA FISH; quantitative analysis of inclusion pathology	*•* Overexpression of poly-GA induced formation of p62-positive neuronal cytoplasmic inclusions in rat primary neurons *•* Overexpressed poly-GR and poly-PR formed nucleolar p62-negative inclusions *•* In C9-ALS patient tissue, neuronal inclusions of poly-GR, -GP and -GA co-localised with Unc119 *•* The authors noticed a correlation between the abundance of poly-GA and Unc119 inclusions and the diagnosis of FTLD vs MND
Sudria-Lopez et al. [100]	*C9orf72* knockout mouse model with full ablation of C9orf72 in all tissues Histopathological analysis	*• C9orf72* knockout mice exhibited decreased body weight, enlarged lymph nodes and splenomegaly *•* Multiple organs in those mice contained macrophage and lymphocyte infiltrates *•* Neoplastic events were also reported *•* No evidence of motor neuron degeneration, gliosis or TDP-43 inclusions
Wen et al. [73]	Rat primary cortical and motor neuron cultures transfected with PR_50_ cDNA and *C9orf72* shRNA constructs; transgenic *Drosophila* model expressing poly-PR_50_; iPSC-derived neurons transfected with PR_50_; spinal cord tissue from C9-ALS/FTD patients IHC; quantification of nucleoli and P-bodies	*•* Due to its intrinsic properties including aggregation in the nucleolus, formation of stress granules and reduction in the number of processing bodies, poly-PR was found to be the most toxic DPR *•* Nuclear aggregates of poly-PR were found in an iPSC-derived motor neuron from C9-ALS/FTD patients and post-mortem spinal cord tissue of patients
Yamakawa et al*.* [75]	Synthetic cDNA encoding 100 repeats of poly-GA, poly-GP, poly-GR, poly-PR and poly-PA, without G_4_C_2_ repeats, was used to study the effects of those DPRs on transfected cultured neuronal cell lines (HeLa and HEK293) in vitro and mouse cortical neurons IHC, IF and immunoblot detection and characterisation of DPRs in cells, in utero electroporation, IHC of brain slices	*•* Out of the five DPRs, poly-GA was found to have the highest tendency to form aggregates and inclusions, in a poly-GA repeat length-dependent manner, in cultured neuronal lines *•* Poly-GA inclusions were p62 and ubiquitin positive but negative for TDP-43 *•* Poly-GR and poly-PR formed ubiquitin- p62-negative cytoplasmic inclusions co-localised with TDP-43 *•* Overexpression of poly-GA, -GP and -GR caused dysregulation of the cellular ubiquitin–proteasome system, which is crucial to protein homeostasis
Zhang et al. [43]	*Drosophila* expressing 30 G_4_C_2_ repeats; S2 cells expressing *Drosophila* RanGAP protein; C9-ALS patient-derived iPSNs; post-mortem brain tissue from *C9orf72* patients Western blot; electrophysiological recording; RNA FISH; IF, IHC and Phalloidin staining; FRAP analysis; electrophoretic mobility shift assays	*•* Candidate-based genetic screen, in *Drosophila* expressing (G_4_C_2_)_30_ repeats, identified RanGAP (orthologue of human RanGAP1 — regulator of nucleocytoplasmic transport) as a potential suppressor of C9-mediated neurodegeneration *•* RanGAP was found to interact with G_4_C_2_ RNA and mislocalise in (G_4_C_2_)_30_ *Drosophila,* iPSNs and brains of C9-ALS cases *•* G_4_C_2_ repeat expansion induced impairment of nuclear import in the fly model and C9-ALS patient-derived iPSNs *•* Small molecules and antisense oligonucleotides targeting G_4_C_2_ repeat expansion G-quadruplex rescued deficits in nuclear import
Zhang et al. [33]	Transgenic mouse model expressing 50 repeats of poly-GA by means of AAV1 viral injection; primary neuronal cultures; HEK293T cells transfected with GFP-(GA)_50_; post-mortem tissue from C9-ALS cases IHC; IF; immunoelectron microscopy; quantification of neuropathology; silver staining; RT-qPCR; co-immunoprecipitation; immunoblotting; mouse behavioural testing	*•* Poly-GA toxicity was accompanied by behavioural abnormalities and neurodegeneration in mice expressing (GA)_50_ *•* Aggregation of poly-GA was required for the manifestation of phenotypes resembling C9-ALS pathology in these mice *•* Poly-GA was found to sequester HR23 proteins which are involved in proteasomal degradation in (GA)_50_ mice *•* HR23A and HR23B co-localised with poly-GA inclusions in post-mortem tissue of C9-ALS cases *•* Aggregation of poly-GA and poly-GA-induced toxicity were attenuated in neuronal cultures when HR23B levels were restored
Zu et al. [12]	HEK293T cells transfected with antisense (G-_2_C_4_)_40/50_; *C9orf72* patient tissue IF, IHC, FISH, Western blot, protein dot blot, cell toxicity and viability assays, RT-PCR	*•* Antisense transcripts of C9orf72 are increased in *C9orf72* patients and accumulate into antisense foci *•* Sense and antisense foci were detectable in the blood acting as a potential biomarker *•* DPRs can also present as antisense giving rise to poly-PR, -PA and -GP. Poly-GP is a repeat as it encoded in the sense direction as well. These accumulate in the frontal and motor cortices as well as the spinal cord and hippocampus

Abbreviations: *ALS*, amyotrophic lateral sclerosis; *ATM*, ataxia telangiectasia mutated; *Cas9*, CRISPR-associated 9; *cDNA*, circular DNA; *CRISPR*, clustered regularly interspaced short palindromic repeats; *DPR*, dipeptide repeat; *ELISA*, enzyme-linked immunosorbent assays; *FISH*, fluorescent in-situ hybridisation; *FRAP*, fluorescence recovery after photobleaching; *FTD*, frontotemporal dementia; *Gal4*, galactose 4; *GFP*, green fluorescent protein; *GMR*, glass multiple reporter; *H&E*, haematoxylin and eosin; *HEK293*, human embryonic kidney cells; *hnRNP*, heterogenous nuclear ribonucleoproteins; *HR23*, UV excision repair protein RAD23 homolog B; *ICC*, immunocytochemistry; *IF*, immunofluorescence; *IHC*, immunohistochemistry; *iPSC*, induced pluripotent cells; *ISH*, in situ hybridisation; *Ku80*, Lupus Ku autoantigen protein p80; *lacZ*, lactose operon Z; *LC3*, microtubule-associated proteins 1A/1B light chain 3B; *LC–MS*, liquid chromatography mass spectrometry; *MND*, motor neuron disease; *NSC-34*, motor neuron-like hybrid line; *p53*, tumour protein 53; *p62*, ubiquitin-binding protein; *PCR*, polymerase chain reaction; *qPCR*, quantitative polymerase chain reaction; *Rab*, ras-associated binding protein; *RNA*, ribonucleic acid; *RNP*, ribonucleoprotein; *RT-PCR*, real-time PCR; *sdRNA*, small self-deliverable interference RNA; *shRNA*, short hairpin RNA; *siRNA*, small interfering RNA; *TDP-43*, tar-DNA binding protein 43; *TM6B*, third chromosome balancer; *UAS*, upstream activation sequence; *Unc119*, uncoordinated 119

In total, 23 studies which specifically referenced the synapse were found, although two of these reported no changes (Table 2). The most frequent finding was that of dendritic arborisation defects (*n* = 8). Moreover, studies reported dysregulation in synaptic genes (*n* = 2), synaptic neurotransmission (*n* = 2) and NMJ morphological changes such as blebbing and loss of synaptic boutons (*n* = 5). Three studies focused on the interaction of *C9orf72* with synaptic proteins, such as the Rab family of GTPases, and two other studies reported axonal degeneration (*n* = 1) and axonal projection impairments (*n* = 1). Of the remaining 50 papers, 32 discussed changes in the cerebellum (Table 3) and the other 18 were relevant publications to *C9orf72* pathology (Table 4).

All human patient data was derived from ALS/FTD cases with a confirmed C9 mutation. The majority of human studies employed histological and/ or molecular analysis of post-mortem tissue (*n* = 45). Publications analysing post-mortem tissue of C9-ALS/FTD cases most commonly reported (i) reduced levels of *C9orf72* proteins (*n* = 5), (ii) DPR aggregates/ toxicity (*n* = 24), (iii) abundant RNA foci (*n* = 10) and (iv) brain region-specific neuronal loss (*n* = 5). There were also case study reports (*n* = 2) demonstrating a link between C9 mutation and cerebellar pathology (cerebellar ataxia and pure cerebellar syndrome) and a large-scale screening clinical study (*n* = 1).

The methodologies used to recapitulate C9-ALS/FTD pathology in vivo and in vitro can be broadly divided into two categories: (i) insertion of G_4_C_2_ repeat expansions of varying length and (ii) expression of DPRs in the absence of G_4_C_2_ repeats. In all selected studies, in vitro data was complemented by post-mortem human data and/or in vivo data. Investigating the contribution of RNA repeats, studies utilised sense and antisense (G_4_C_2_)_n_ expression vectors (*n* = 6) which were used to transfect cell culture lines and study the formation of RNA foci and DPRs. In vitro transfection of DPRs was even more frequently used (*n* = 13). Alternatively, ten studies used *C9orf72*-ALS/FTD patient iPSC-derived neurons. In contrast, a model composed of 100 synthetic cDNA encoded repeats of the five main DPRs — GA, PA, PR, GP and GR — was used to study the effect of those DPRs on transfected cells and primary neuronal cultures*.*

Transgenic mouse lines were the most commonly used in vivo (*n* = 17) with models generated to contain G_4_C_2_ repeats (*n* = 3), express DPR proteins (*n* = 9) or knockout the *C9orf72* gene (*n* = 5). G_4_C_2_ repeats and DPR models were created by means of AAV viral injections. Viral transduction was achieved in the CNS through different promotors such as the cyan fluorescent protein (CFP)-GA_149_ line which expressed DPRs under the control of the Thy1 promotor, ensuring neuron-specific expression. Alternatively, DPR-Nestin lines were generated to drive ubiquitous CNS expression. Of the transgenic mouse lines used to study DPRs, poly-GA (*n* = 4) and poly-PR (*n* = 4) were the most common models followed by poly-GR (*n* = 1). *Drosophila* models were also frequent throughout the studies (*n* = 11). Transgenic fly lines were generated using traditional crossing methods to investigate DPRs (*n* = 7) and G_4_C_2_ repeat-mediated (*n* = 6) pathology. *Drosophila* lines expressing poly-GR (*n* = 6) were the most common, followed by poly-PR (*n* = 5), poly-PA (*n* = 3) and poly-GA (*n* = 2). Zebrafish models (*n* = 3) were generated to study the effect of reduced *C9orf72* protein expression by injection of antisense oligonucleotides (*n* = 1), transient expression of DPRs (*n* = 1) and (G_4_C_2_)_n_ repeats (*n* = 1). Additionally, a *C*. *elegans* model expressing 50 repeats of -GA, -PA, -GR or -PR DPRs (*n* = 1) and two chick embryo models expressing DPRs and G_4_C_2_ repeats were used.

### Meta-Analyses

Of the included papers, meta-analysis was conducted on dendritic arborisation defects (*n* = 8) with this being refined to dendritic abnormalities (*n* = 5) and neurite length (*n* = 3) as two separate analyses and NMJ abnormalities (*n* = 5). Furthermore, reductions in *C9orf72* protein (*n* = 4), cerebellar- and DPR-related neuronal loss were also conducted (*n* = 4 and *n* = 5, respectively). When analysing articles, we also found that many studies reported results that would fit into the previously mentioned categorisations; however, frequently, the data reported was visual (i.e. immunofluorescent imaging) without any quantitative data supporting gain/loss or no effect of each analysis and was therefore excluded.

**Fig. 4:**
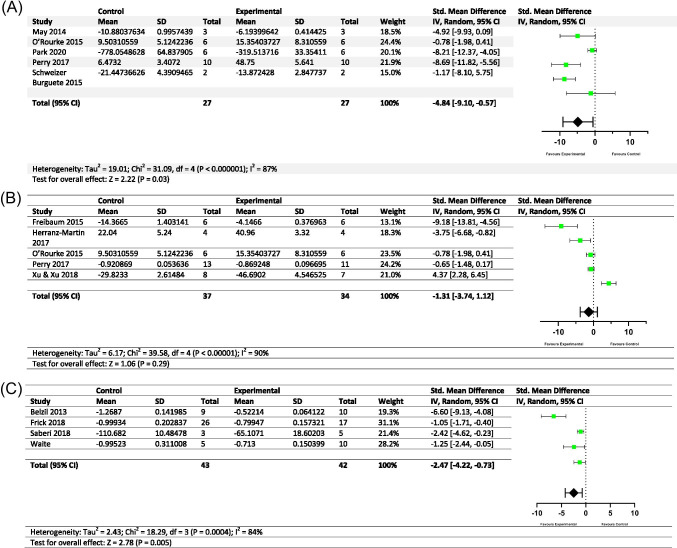
Meta-analysis using a random effects model of selected studies relating to synaptic deficits. (**A**) Shows the meta-analysis for dendritic defects assessing reductions in arborisations such as crossings and branchpoints (*P* = 0.03). (**B**) Shows the meta-analysis for neuromuscular junction (NMJs) abnormalities assessing synaptic bouton counts and fractured NMJs (*P* = 0.29). (**C**) Shows the meta-analysis for reductions of C9orf72 protein in human patients using frontal cortex and cerebellar samples (*P* = 0.005). All studies were highly heterogenous (*I*^2^ ≥ 84%; *P* ≤ 0.0004). The figure was generated using the RevMan 5.4 software. Abbreviations: *SD*, standard deviation; *CI*, confidence interval

**Fig. 5:**
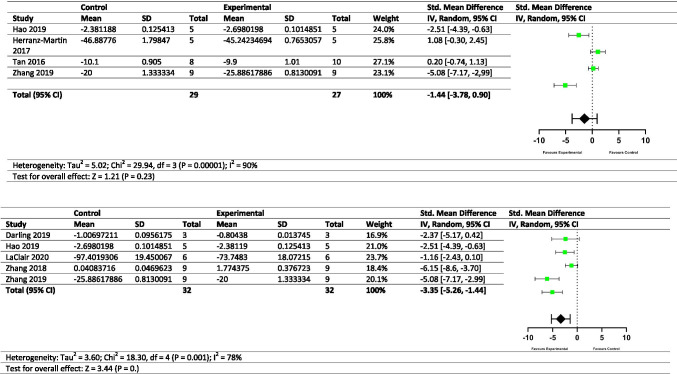
Meta-analysis using a random effects model of selected studies relating to neuronal loss in *C9orf72*. (**A**) Shows the meta-analysis of neuronal loss in the cerebellum as a result of G_4_C_2_ repeats and DPR models (*P* = 0.23). (**B**) Shows the meta-analysis of DPR-related neuronal loss using animal models transfected with DPR constructs (*P* = 0.0006). Studies were highly heterogenous (*I*^2^ $\ge$ 78%; *P* ≤ 0.001). The figure was generated using RevMan 5.4. Abbreviations: *SD*, standard deviation; *CI*, confidence interval

We found that in all meta-analyses (Figs. 4 and 5), the studies were highly heterogenous (*I*^2^ > 75%; *P* ≤ 0.001), most likely a result of different species, repeat lengths, DPR models and other variables changing in each study. Nevertheless, significant dendritic abnormalities were seen in *C9orf72* models of disease (*P* = 0.03) as well as reductions in *C9orf72* protein in human patients (*P* = 0.005) and DPR-related neuronal loss (*P* = 0.0006). Whereas NMJ abnormalities and cerebellar neuronal loss failed to reach significance (*P* = 0.29 and *P* = 0.23, respectively). Moreover, neurite length data was extracted with the intent to analyse; however, the required study size (*n* = 3) was not reached. Therefore, we have included the already extracted data values in Table 5 to be used in future meta-analyses.

**Table 5:** Data table for neurite length meta-analysis

Study or subgroup	Control			Experimental				Std. mean difference IV, random, 95% CI
	Mean	SD	Total	Mean	SD	Total	Weight	
Park 2020	− 21,200.42	688.9353	6	− 9,966.0752	806.3674322	6	43.7%	− 13.83 [− 20.67, − 6.98]
Swaminathan 2018	− 195.9627	8.074534	7	− 161.95652	5.74534162	7	56.3%	− 4.54 [− 6.79, − 2.30]
Zhang 2014	− 266.422	17.61468	0	− 125.77982	20.36697248	0		Not estimable
Total (95% CI)			13			13	100.0%	− 8.60 [− 17.63, 0.43]

Incomplete meta-analysis of neurite length due to Zhang et al. (2014) not having available sample sizes thus an appropriate study size (*N* = 3) was not reached. The table was produced in RevMan 5.4 with the associated graph, and statistical measures were removed from the image. Abbreviations: *SD*, standard deviation; *CI*, confidence interval

## Discussion

Synaptic dysfunction is a common feature in neurodegenerative disease which represents an early disease event taking place before the development of neuronal degeneration and loss [5, 21, 41–45]. Freibaum et al. (2015) [42] assessed the impact of *C9orf72* repeat pathology in *Drosophila* larvae, showing not only a dramatic loss in synapse structure, with severe reductions found at presynaptic active zones [42, 43], but also a significant reduction in synaptic bouton counts [42, 44] and synaptic quantal content [43, 44].

Interestingly, a meta-analysis conducted to assess the state of NMJs (Fig. 4B) found no significant alterations in bouton counts and NMJ integrity despite the reports from Freibaum et al. (2015) [32, 42]. This may be explained by Xu and Xu [21] finding increased synaptic bouton counts whilst the remaining studies found the opposite. Given that an increase in synaptic bouton count could be a result of excitotoxic mechanisms which Xu and Xu themselves report, this may then skew the meta-analysis, as this statistical test is not sensitive to phenomena in which both an increase and decrease in synaptic boutons may represent pathological changes. Indeed, the presence of excitotoxic mechanisms has been reported by other studies [4, 39] in the early stages of the disease. Devlin et al. (2014) [41] demonstrated that *C9orf72* patient iPSC-derived motor neurons (MNs) revealed hyperexcitability at early stages in culture followed by a progressive loss of action potential output, a finding previously reported in other animal models of ALS [46, 47]. This initial phase of increased activity has been suggested to trigger a cascade of excitotoxic disease mechanisms involving pathological changes in Ca^2+^ handling [48, 49], accumulation of intracellular Ca^2+^ and the eventual activation of cell death pathways. Other early events observed by the authors were the loss of synaptic activity in *C9orf72*-iPSC-derived MNs which could be reflecting a general loss of action potential generation in culture; however, due to the evidence of loss and dysfunction of synapses in ALS [50–55], specific deficits in synaptic transmission might also contribute to reductions in synaptic activity recorded from patient iPSC-derived MNs. Indeed, Jensen et al*.* (2020) [4] found that the presence of poly-GA results in hyperexcitability, through Ca^2+^ depolarisation, of neurons followed by a reduction in SV2 — mirroring Devlin et al.’s [41] results of the initial appearance of excitotoxic mechanisms followed by a loss of synaptic activity, or in this case, an inability to unload the synaptic vesicles properly. Moreover, the animal model used by Jensen et al. [4] exclusively expressed poly-GA in the spinal cord, brainstem and cerebellum before any motor neuron loss was observed. This resulted in time-dependent and poly-GA-dependent gait and behavioural deficits [4], possibly suggesting that SV2 and neuronal excitability are more widespread than just MNs as Devlin et al. [41] reported and may include any neuron which harbours poly-GA expression. Devlin et al*.* also demonstrated changes in ionic conductance which could indicate that early dysfunction or loss of ion channels may contribute to the initiation of downstream degenerative pathways that ultimately lead to MN loss in ALS [41]. This ‘functional loss’ of neurons may render the motor system, including the cerebellum, inactive before neurodegeneration occurs. Both papers highlight the importance of understanding synaptic pathology as this may precede and promote neuronal death. Therefore, they present viable therapeutic options to prevent neurodegeneration from occurring rather than attempting to rescue already dying tissues.

Moreover, the dysregulation in synaptic transmission in the motor system was also corroborated by Hao et al*.* (2019) [56]. In their study, they expressed 28 × poly-PR (GFP-PR_28_) under the control of the neuronal *Thy1* promoter, and they found that heterozygous mice developed deficiency of motor performance at 6 months of age. These motor deficits were accompanied by cerebellar poly-PR inclusions together with increased activation of astrocytes and microglia in the cerebellum and spinal cord, whereas only a few astrocytes in the motor cortex and no gliosis in the hippocampus were observed in these animals. Moreover, the GFP-PR_28_ heterozygous mice showed atrophy of the cerebral cortex and loss of Purkinje cells in the cerebellum and motor neurons in the spinal cord. These results are in line with the greater cerebellar atrophy showed by Gendron et al. (2013) [27] in *C9orf72*-FTD patients, highlighting the direct relationship between the *C9orf72* mutation and cerebellar and motor defects. Moreover, other subtypes of ALS such as SOD1 and MAPT have shown cerebellar pathology [57], revealing the relevance of this brain region in ALS pathology. Finally, we wanted to note that although cerebellar ataxia is not typically observed in other types of FTD [58–60], Fogel et al. (2012) [56] have found a case where cerebellar ataxia was identified in *C9orf72*-ALS/FTD patients.

### Understanding the Role of DPRs in C9-Cerebellar Pathology

Moreover, although the evidence has pointed out that the expression of DPRs is one of the causative factors for *C9orf72*-ALS/FTD based on the identification of DPRs in patient brains [12, 26, 29, 33, 61–68] and expression of DPRs without hexanucleotide-repeat RNA in animal models [33, 64], it is unknown whether DPR localisation correlates to neuronal loss. In order to elucidate this, Zhang et al. (2019) [69] generated a mouse model through viral infection of AAV1 GFP-PR_50_. This resulted in motor dysfunction and cognitive deficits, with reduced brain weight and an age-dependent loss of poly-PR expressing Purkinje cells and cortical neurons at 3 months of age, suggesting that poly-PR expression caused cell-autonomous neuron death. These pathological hallmarks were also accompanied by increased astrogliosis and microgliosis in the cortex and cerebellum of GFP-PR_50_ animals. Moreover, a poly-GR_100_ AAV-infected animal model also showed cortical thinning, hippocampal cell loss and cerebellar Purkinje cell loss from 1.5 to 6 months of age, whereas no spinal cord neuron loss was seen. These changes were parallel to increased astrogliosis and microgliosis from 1.5 months in GFP-GR_100_ mice and locomotor impairment [65]. Both studies not only highlight the impact of arginine enriched DPRs on the cerebellum and motor function, but also the correlation between DPR length and onset of pathogenesis, with shorter lengths of poly-PR having a later onset of age-related deficits [56, 69].

Indeed, our meta-analysis provides evidence linking DPRs with neuronal loss in C9-ALS/FTD (Fig. 5B). However, the effect DPRs have in the cerebellum specifically is difficult to assess due to multiple brain regions being pooled for this meta-analysis in order to reach an appropriate study number. In fact, when we assessed neuronal loss in the cerebellum, regardless of DPR involvement, we found no significant reduction in the Purkinje cells (Fig. 5A). This may be due to the fact that different areas in the cerebellum may be more prone to degeneration such as the spinocerebellum compared to the lateral cerebellar hemispheres [70]. Therefore, it is important for studies to report which specific subregion was used to carry out quantification to allow for more accurate pooling of data which may lead to more meaningful conclusions from a meta-analysis in the future.

Another important finding in these studies is that different DPRs interfere with different cell processes. The gene ontology (GO) analyses after RNA sequencing in the GFP-PR_28_ heterozygous mice model used by Hao et al. (2019) [56] revealed dysregulation of synaptic transmission-related genes, specifically downregulated genes included calcium ion-regulated exocytosis of neurotransmitters, intracellular signal transduction and neurotransmitter secretion. These results point out that transmission across chemical synapses is a major pathway implicated in PR_28_ cerebellar pathology. They have also found that two genes (*Rims3*, *Doc2b*) related to synaptic function were downregulated in the cerebellum of 2-month-old heterozygous mice. Moreover, upregulation of ER-stress genes (*Chac1* and *Atf5*) has been found in the cerebellum of 2-month- and 5- month-old heterozygous mice, suggesting that ER stress and synaptic dysfunction in the cerebellum are early events in poly-PR expressing neurons.

However, the GO analyses in the GFP-GR_100_ mice reported by Zhang et al. (2018) [65] did not show synaptic-related differences but ribosomal and protein translational changes. These results are opposite to the studies where the accumulation of arginine-rich DPRs (GR and PR) in the nuclei of many cell types [11, 63, 71, 72] have suggested that they could be the cause of the dendritic defects observed, which is thought to precede neuronal cell death in ALS-FTD [24]. Park et al. (2020), using a *C9orf72 Drosophila* model, proved that arginine-rich DPRs led to the most significant reduction in dendritic branches and reduced the number of Golgi outposts in dendrites, which are organelles with a role in the cytoarchitecture of neuronal dendrites, leading to defective vesicular trafficking in dendrites. Moreover, Swaminathan et al*.* (2018) show that [73] poly-GR_100_ was capable of reducing motor neuron length in a zebrafish model [74]. Despite the methodological heterogeneity, our meta-analysis found significant alterations in dendritic branching, further giving evidence that synaptic dysfunction is a feature of C9-ALS/FTD (Fig. 4A). These results encourage further research into the cerebellum where early pathological features are detected. Most importantly, these studies compile evidence that the accumulation of different DPRs in different brain regions during the progression of the disease could explain the dynamics contributing to the neurodegeneration seen in *C9orf72*-ALS/FTD patients.

### The Relevance of DPR Dynamics in C9-Cerebellar Pathology

A clear example of the need to study DPR dynamics during the cerebellar progression of the disease is represented by Yamakawa et al. (2015) in the brains of patients with *C9orf72*-ALS/FTD [75]. The authors found that the five DPRs (poly-GA, poly-GP, poly-GR, poly-PR and poly-PA) are deposited in the granular neurons of the cerebellum as reported by others [25, 76]. Further to this, Mackenzie et al. (2015) found that poly-GA inclusions (sense transcript) were by far the most abundant, followed by poly-GP (sense and antisense transcripts) and poly-GR inclusions (sense transcript) with only rare poly-PR and poly-PA inclusions (antisense transcripts) throughout the premotor frontal cortex, lower spinal cord MNs and cerebellum [26]. These data, as well as early-onset DPR inclusions in animal models and their inherent toxicity, illustrate the importance of studying the accumulation of brain DPRs in a time- and cell-dependent fashion and may explain why some poly-DPR pathologies are rare in post-mortem brain tissues from *C9orf72*-ALS/FTD patients, which reflect the end stage of disease and may likely have neuronal death obscuring the mechanisms through which DPRs are causing toxicity.

The toxicity of DPRs may also be related to and depend on each other. Zhang et al*.* (2018) [65] confirmed a previous report by Yang et al. (2015) [77] that poly-GR only aggregates in cells that have insoluble poly-GA aggregates and remains diffuse without the presence of poly-GA. Interestingly, Yang et al. found that poly-GA recruiting poly-GR to aggregates was found to prevent certain measures of toxicity and restore defective Notch signalling, suggesting that poly-GA, which readily aggregates, may provide some defence mechanism against toxicity [77]. However, it was revealed that poly-GR was still able to interact with ribosomal/mitochondrial targets when aggregated [65]. The ability to do so may impact the dynamics within the cell such that aggregated poly-GR interacting and binding to targets may impair nearby reactions due to steric hindrance.

Moreover, poly-GA’s ability to alter DPR chemistry is not limited to poly-GR. Darling et al. (2019) co-expressed poly-GA with poly-PR and interestingly found that high levels of poly-GA in relation to poly-PR — ratios of 10:1 and 5:1 — localised poly-PR to the cytoplasm, with the nucleus being the typical aggregate locale of poly-PR [78]. Additionally, it was found that co-expression of poly-GA_50_ reduced aberrant phosphorylation in the unfolded protein response — a characteristic of poly-PR aggregates — preventing potential triggering of an apoptotic event through the PERK pathway. However, there is some controversy regarding the roles of DPR synergy in causing toxicity as Lee et al. (2017) found that poly-GA did not co-localise frequently with poly-GR and -PR [39]. Although this could be explained by poly-GR and -PR being much rarer in the brain in general, they demonstrated that poly-GA could sequester poly-GP and -PA, but, in their model, sequestration of poly-PA with poly-GA actually prevented GA-mediated toxicity and not GA-mediated reduction of toxicity of the other DPRs as seen by Zhang and Yang (2018; 2015) [65, 77]. Nevertheless, the ability for DPRs to interact with one another and alter their toxicity suggests that structures which express all variants of DPRs such as the cerebellum will likely have different alterations to metabolic pathways which could explain differences from cortical and motor neuron degeneration along the life span of *C9orf72* patients [39, 65, 75, 77].

### Pathways Mediating DPRs Toxicity in C9-Cerebellar Pathology

Another important hallmark of the cerebellum in *C9orf72* mutation carriers is the presence of TDP-43-negative and p62-positive neuronal cytoplasmic inclusions [22, 25, 79, 80] which have been shown to be associated with poly-GA, GP, GR, PA and PR [11, 22, 30, 31, 61, 81]. In this regard, Mann (2013) observed a correlation between p62 inclusions and poly-GA and poly-GP in the cerebellum [31]. Moreover, in the study, a correlation between poly-GA and -GP, together with poly-GR and -PR, was also demonstrated. Although the role of poly-PR was recently established by Maor-Nof et al. (2021) [82] and Far and Shorter (2021) [83] as a remodeller of the neuronal epigenome to promote proapoptotic p53 activity involving PUMA, the inconsistency of its presence in all expansion bearers of the Mann study (2013) [31] casts doubt into its relevance to cerebellar pathology. Despite this, Maor-Nof’s study [82] proposed for the first time a relationship between poly-PR and axonal degeneration that could be rescued by p53 reduction, increasing survival of *C9orf72*-ALS/FTD patient-induced pluripotent stem cell (iPSC)-derived motor neurons. Moreover, we cannot rule out that poly-PR and its inconsistent presence may be a result of the toxicity it exerts on the neuron, such that all neurons which are PR positive die earlier than neurons which lack PR inclusions or have a low poly-PR burden.

Furthermore, Lopez-Gonzalez et al. also found that poly-GR increases p53 levels in neurons of *C9orf72* patients, and axonal degeneration may be a unique pathology associated with arginine-DPRs [84]. Mori et al. (2013b) demonstrated a rostro-caudal gradient of poly-GR inclusions being abundant in all neocortical areas, hippocampus and cerebellum, with moderate abundance in subcortical nuclei and low abundance in the brain stem and spinal cord which may highlight areas particularly susceptible to poly-GR-mediated degeneration [85]. However, in these studies, the contribution of these DPR inclusions to cerebellar synaptic function and the pathways potentially involved has been scarcely studied. Despite this, these results shed light on the relationship between DPRs and downstream degenerative cascades that could be targeted at early stages in specific brain regions. Moreover, p53 cerebellar reduction in relation to poly-PR and poly-GR accumulation in *C9orf72* ALS/FTD-patient remains unclear, but further research may highlight potential therapies which could be applicable to other diseases such as spinal muscular atrophy and Purkinje cell degeneration [86, 87].

In a poly-GA mouse model, LaClair et al. (2020) demonstrated that poly-GA has a greater propensity to aggregate in the cerebellum which may drive toxicity seen in these mice [81]. These results are also supported by the increased number of inclusions found in the molecular and granular layers of the cerebellum of *C9orf72* mutation cases by Mori et al. (2013b), whereas inclusions were rarely found in the Purkinje layer [85]. Moreover, LaClair et al. (2020) [81] have shown that poly-GA promotes interferon responses in *C9orf72* disease and contributes to TDP-43 abnormalities and neuron loss selectively in disease-relevant regions.

An important study from May et al. (2014) [22] showed that poly-GA co-localised with Unc119, a transport factor previously linked to neuromuscular and axonal function, in the cerebellum. In the study, it was demonstrated that similar to poly-GA expression, Unc119 knockdown inhibits dendritic branching and causes neurotoxicity, suggesting that poly-GA expression may be the driving force for Unc119 aggregation. Interestingly, those who present with *C9orf72*-FTD have higher aggregated levels of poly-GA and Unc119 in the cerebellum than those of *C9orf72*-ALS. However, Schludi et al. (2017) [88] have shown that despite eliciting behavioural deficits and inflammation, no neuronal loss was seen in mouse models expressing poly-GA, suggesting that poly-GA may be instrumental in protein sequestration [89] and inflammation of ALS but not end-stage neuronal death. Jensen et al. (2020) [4] further support this intermediary role of poly-GA in reporting aberrant synaptic unloading and motor deficits without overt neuronal death, as we have previously discussed.

### The Role of DPR Solubility in C9-Cerebellar Pathology

Despite the overwhelming evidence of DPR aggregates in C9-ALS/FTD, it is unclear whether binding DPRs to insoluble aggregates causes toxicity or whether the soluble form of DPRs is toxic and aggregation is a defence mechanism [30, 90]. In this regard, Quaegebeur et al. (2020) demonstrated differing solubility profiles of DPRs across human brain tissue [91]. Notably, soluble DPRs tend to be less abundant in areas associated with clinical pathologies, such as the frontal cortex in FTD. Interestingly, the cerebellum has significantly different dynamics from the other cortices measured. Despite the aggregate load of poly-GA and -GR being comparable to the frontal and temporal cortices, insoluble poly-GP was highest in the cerebellum. Moreover, the soluble forms of poly-GA and -GP are at much greater concentrations in the cerebellum than other regions, with poly-GR showing significant variability between all cortices. With Quaegebeur et al. (2020) reporting that reductions in soluble fractions were associated with disease severity [91], one would likely presume that this is because they are aggregating. Therefore, it is surprising that the cerebellum, which has higher levels of soluble and insoluble DPRs, shows less neurodegeneration when compared to the frontal cortex. This may indicate a mechanism by which the toxicity of DPR aggregation is in some way mitigated in the cerebellum and is seen as ‘spared’ in *C9orf72*-ALS/FTD. These regional differences in DPR solubility could be pointing out the selective vulnerability of different brain regions to DPRs, underlined by different degradation pathways along the brain. Furthermore, this may mean the cerebellum may present as the most useful structure to investigate the effect of synaptopathy and of the macro-mechanisms of reduced *C9orf72* protein and RNA foci due to the mitigation of DPR-dependent toxicity as well as investigating solubility mechanisms and its regulation.

### Reduced C9orf72 Protein Show Toxicity Through Downstream Effects

As a consequence of *C9orf72* repeat expansions, *C9orf72* protein levels are reduced, and the resulting loss of function is one of the key patho-mechanisms of the disease. As we have previously mentioned, the cerebellum is a brain region known to express high levels of *C9orf72* mRNA [2], and differences in cerebellar transcript levels between *C9orf72* mutations carriers and controls [92, 93] have also been found. Frick et al. (2018) confirmed previous reports of reduced *C9orf72* protein levels in the cerebellum of *C9orf72* mutation carriers with no association to clinical phenotypes (ALS, ALS/FTD or FTD), age at onset and disease duration [16]. The authors explained this finding by a strong positive correlation between the presence of neurodegeneration/cell death and *C9orf72* levels in cerebellar regions. Similar results in post-mortem tissues were reported by Tan et al. (2016) [70] where no neuronal loss was identified in the cerebellum. However, opposite results were found by Chew et al. (2015) in which mouse models of (G_4_C_2_)_66_ showed loss of Purkinje cells [94]. Similar results were found by Liu et al. (2016) in a BAC mouse model of *C9orf72* [95], so Frick’s [16] results might be potentially explained by protein degradation due to post-mortem delay in human samples. Moreover, Saberi et al. (2017) [96] and Waite et al. (2014) [93] found reduced *C9orf72* protein expression in frontal areas but not in the cerebellum. Nevertheless, performing a meta-analysis (Fig. 4C) which combined frontal and cerebellar brain regions found that *C9orf72* levels are significantly reduced in human patients at the time of autopsy.

It is also important to note that the mechanisms by which reduced protein levels contribute to *C9orf72* disease pathogenesis are still unknown and may not be causing neurodegeneration per se. This is evident with reductions in *C9orf72* being present in brain regions affected by neurodegeneration and those spared from it. Moreover, the lack of clinical phenotypes in *C9orf72* knockout mice seems to support this view [97–100]. However, Frick et al. (2018) identified *C9orf72* protein to be localised presynaptically and able to interact with members of the RAB3 protein family, suggestive of a role for *C9orf72* in regulating stress vesicles function by potentially acting as guanine nucleotide exchange factors (GEFs) for specific Rab GTPases (Rabs) such as RAB3 [16]. These results could support a role for cerebellar *C9orf72* in regulating synaptic vesicle function.

**Fig. 6:**
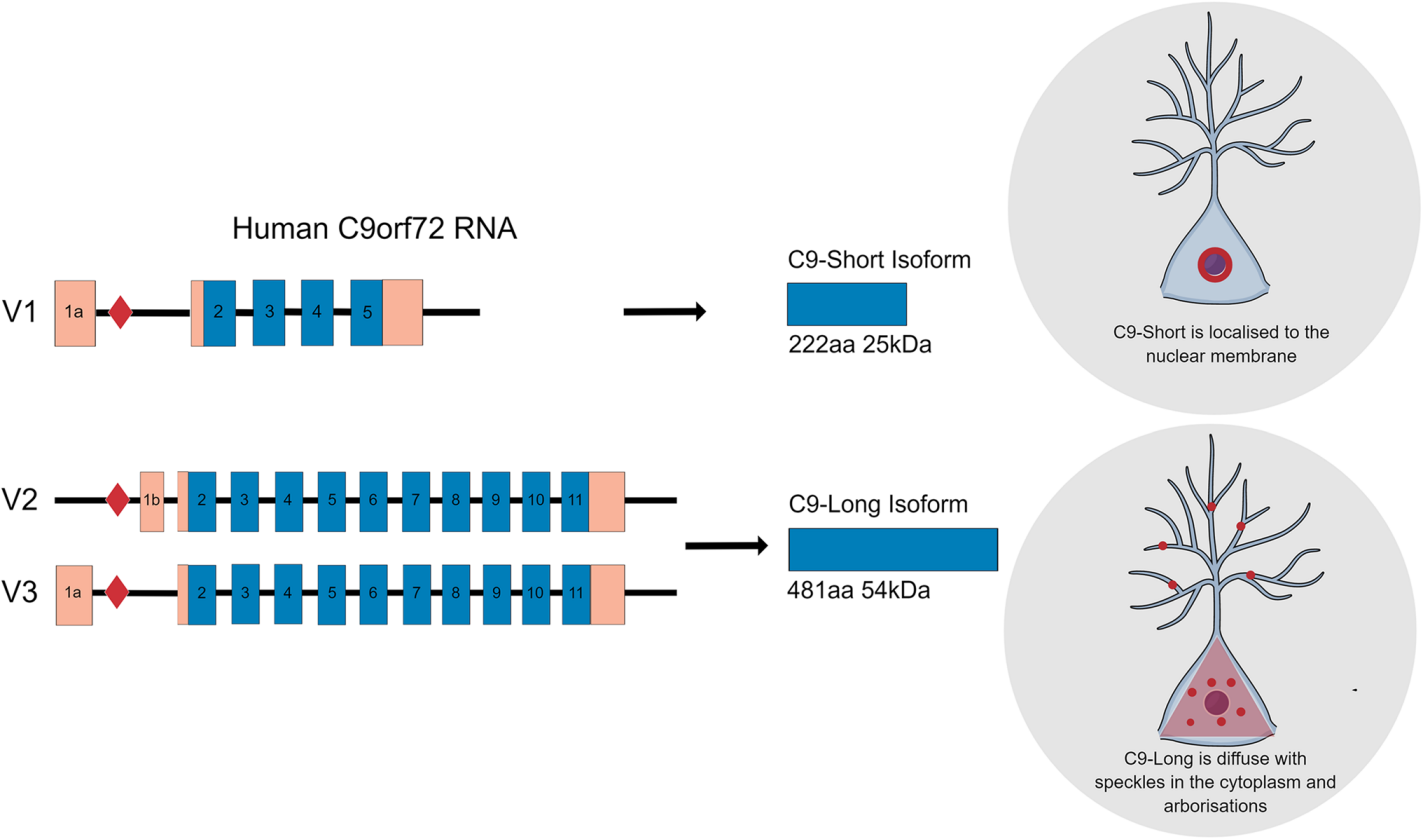
Splice variants of human *C9orf72* mRNA. An overview of the exons included in the long and short isoforms of C9orf72 and their subcellular localisation. Abbreviations: *aa*, amino acids; *kDa*, kilodalton; *RNA*, ribonucleic acid; *V1/2/3*, variant 1/2/3

Another factor that could be contributing to contradictory results regarding the precise functions and properties of *C9orf72* protein is the lack of specific antibodies [101, 102]. In this regard, it is important to mention that *C9orf72* generates three transcripts through alternative splicing that encode 2 protein isoforms: a long isoform of approximately 54 kDa (termed C9-L), corresponding to variants 2 (V2) and 3 (V3) and a short isoform of approximately 24 kDa (termed C9-S), corresponding to variant 1 (V1) (overviewed in Fig. 6) [103]. Haploinsufficiency was initially suggested as a disease mechanism owing to the decreased abundance of V2 and V3 transcripts in *C9orf72-*ALS cases, leaving the contribution of C9-S to the disease unknown. Xiao et al. (2015) reported two antibodies capable of detecting both C9 isoforms [104]. In their study, C9-L showed diffuse labelling in the cytoplasm of cerebellar Purkinje cells, with a striking labelling of numerous speckles that were observed in both the neuronal perikarya and dendritic processes in *C9orf72* carriers and non-carriers. In contrast, the C9-S antibody gave a very specific labelling of the nuclear membrane. These data showed that C9-L and C9-S have different subcellular localisations in Purkinje cells and suggest that *C9orf72* proteins could play a role in the disruption of the nucleocytoplasmic transport. Davidson et al. (2018) later expanded on this to include commercial antibodies, and whilst some could recapitulate the staining of C9-L, none showed similar staining to C9-S which remains elusive [105].

Recent evidence suggests that *C9orf72* protein complexes with p62 and may lead to pathology through inclusion formation [106]. P62-positive inclusions remain a hallmark of C9-ALS/FTD; however, little research has been conducted into the side effects of the aggregated protein, and there remains a large gap in the current literature [22, 25, 79, 80]. Having reduced *C9orf72* protein, p62 accumulates with additional symmetrical methylated arginine proteins ordinarily used in the complex with *C9orf72* [106]. Bieniek et al. (2013) suggest that aggregation of p62 means there is less to bind to tau resulting in build-up of hyperphosphorylated tau and axonopathy [107]. Moreover, as we have previously discussed, the co-localisation of p62 to DPRs, specifically poly-GA which sequesters Unc119 [22], highlights the complex and potentially synergistic effects of both reduction in *C9orf72* protein levels and DPR expression in dendritic and axonopathy and the pathogenesis of C9-ALS/FTD [11, 22, 30, 31, 81, 85].

### RNA foci

Finally, we also want to discuss the role of intranuclear neuronal RNA foci containing G_4_C_2_ repeats in cerebellar ALS and FTD tissues with RNA foci accumulation being reported by several authors [27, 36, 37, 108]. Indeed, Lee et al. (2013) have found discrete intranuclear neuronal RNA foci where larger RNA foci were found in the cerebellum (500 nm) compared to the cortex (200 nm), which were most frequent in neurons adjacent to Purkinje cells [36]. Also, the authors observed co-localisation between the foci and hnRNP-H, suggesting that sequestration of hnRNP-H itself, other RNA-binding protein and multiple RNA transcripts could be leading to significant dysregulation of RNA processing and toxicity in the cerebellum. Similar results were found by Cooper-Knock et al. (2014) where co-localisation of RNA foci and hnRNP in cerebellar granule cells were found [109]. More recently, Mehta et al. (2020) found evidence of abundant RNA foci in all cell types of the cerebellum without concomitant TDP-43 pathology in *C9orf72* post-mortem tissue whilst using a high-resolution modified in situ hybridisation technique, BaseScope™ [110]. Moreover, an AAV9-mediated expression of (G_4_C_2_)_102_ repeats in mice leads to increased number of RNA foci in the Purkinje cell layer of the cerebellum at 12 months after AAV delivery with increased apoptotic markers and infrequent TDP-43 aggregates [111]. These results are opposite to the correlation identified between antisense RNA foci and TDP-43 pathology in motor neurons of *C9orf72* patients [112], where the authors suggested that RNA foci may be a cause of TDP-43 inclusions.

Neuritic RNA foci are not a commonly discussed feature of *C9orf72*, usually focusing on intranuclear inclusions. However, Schweizer Burguete et al. (2015) found that approximately 80% of neurons with intranuclear inclusions also presented neuritic foci [23]. Most importantly, neuritic RNA foci were found to reduce primary dendritic branching by up to 50% which was not a result of branching capability being hampered. Indeed, early fly larvae exhibited normal branching morphologies; however, upon body growth within the same instar stage, these dendrites failed to extend to cover the increased brain expanse, thereby indicating that neuritic foci disrupt the dendrite’s ability to extend its branches during growth and may indicate early-stage synaptopathies in *C9orf72* carriers during development. Moreover, they found that neuritic-localised foci were the only RNA foci to elicit such branching defects with somatic or nuclear inclusions not impacting dendritic arborisation. Supporting this, foci were bidirectionally transported throughout the cell through mRNP transport vesicles, and this was found to directly impact morphological features of the dendrites. In neurons with a knockdown of FMRP (fragile X mental retardation protein) and other transport-associated proteins, dendritic pathology was attenuated as foci could no longer move from proximal–somatic areas to neuritic–distal locales of the neuron. The concomitance of nuclear foci and neuritic foci suggests a role in the cerebellum which has high foci pathology; however, the exact abundance of neuritic foci is yet to be explored in these areas and therefore may help elucidate the state of dendritic processes and ultimately, synaptic dysfunction, within the cerebellum. Indeed, special attention should be given to understanding the dynamics of differentially localised RNA foci in relation to cerebellar synaptic dysfunction and other deleterious effects their localisation may result in.

## Conclusion

Collectively, the data assembled through this review provides clear evidence that RNA foci and proteinaceous inclusions contribute to synaptic deficits and cerebellar neurodegeneration and should be considered characteristic features of C9-ALS/FTD. This review highlights the relevance of the cerebellum in understanding *C9orf72* pathology and may act as a unique structure to understand synaptic pathology which, to date, has been largely neglected. Special attention should be given to cerebellar pathology not only at early stages, but also throughout the course of the disease, which could shed light on some assumptions regarding the combined actions of reductions in *C9orf72* protein, RNA foci and DPRs as contributing factors to C9 synaptopathy.

## Data Availability

All data generated or analysed during this study are included in this published article. Figures 1 and 6 were created using the online platform MindtheGraph, with the authors possessing full authorial rights.
